# The impact on functioning of second-generation antipsychotic medication side effects for patients with schizophrenia: a worldwide, cross-sectional, web-based survey

**DOI:** 10.1186/s12991-020-00292-5

**Published:** 2020-07-13

**Authors:** Rajiv Tandon, William R. Lenderking, Catherine Weiss, Huda Shalhoub, Carla Dias Barbosa, Jun Chen, Mallik Greene, Stine R. Meehan, Laëtitia Bouérat Duvold, Celso Arango, Ofer Agid, David Castle

**Affiliations:** 1 Western Michigan University Homer Stryker M.D. School of Medicine, 300 Portage Street, Kalamazoo, MI 49007 USA; 2 Evidera, 7101 Wisconsin Avenue, Suite 1400, Bethesda, MD 20814 USA; 3 Otsuka Pharmaceutical Development & Commercialization, Inc., 508 Carnegie Center Drive, Princeton, NJ 08540 USA; 4 H. Lundbeck A/S, Ottiliavej 9, 2500 Valby, Denmark; 5 Department of Child and Adolescent Psychiatry, Institute of Psychiatry and Mental Health, Hospital General Universitario Gregorio Marañón, IiSGM,CIBERSAM, School of Medicine, Universidad Complutense, Calle del Dr. Esquerdo, 46, 28007 Madrid, Spain; 6 Schizophrenia Division, Complex Care & Recovery Program, Centre for Addiction and Mental Health, Toronto, Canada; 7 Department of Psychiatry and Institute of Medical Science, University of Toronto, 1001 Queen Street West, Toronto, ON M6J 1H4 Canada; 8 St Vincent’s Health and The University of Melbourne, 41 Victoria Parade, Fitzroy, VIC 3065 Australia

**Keywords:** Schizophrenia, Web survey, Second-generation antipsychotics, Medication, Side effects, Functioning, Patient-centered, Impacts

## Abstract

**Background:**

It is well established that the different antipsychotics used for schizophrenia symptoms differ substantially in their side effects. However, relatively little is known about the impact of these side effects on functioning from the patient’s perspective. We aimed to understand how key side effects of second-generation antipsychotics impact the functioning and quality of life (QoL) of patients with schizophrenia.

**Methods:**

This is a cross-sectional, web-based survey of patient-reported side effect burden of antipsychotic drugs in adults with schizophrenia. The survey was deployed in the United States, Canada, Australia, Spain, Italy, Norway, and Denmark. It included sociodemographic and clinical questions, the Quality of Life Enjoyment and Satisfaction Questionnaire Short Form (Q-LES-Q-SF), and the Glasgow Antipsychotic Side-Effect Scale (GASS). Eight pre-defined *key* side effects classified as *activating* (“Shaky hands or arms,” “Restlessness,” and “Difficulty sleeping”), *sedating* (“Sleepy during the day”, “Feeling drugged or like a zombie,” and “Feeling dizzy/Fainted”) or *other* side effects (“Problems enjoying sex” and “Gaining weight”), and additional questions related to impacts on function and quality of life were asked.

**Results:**

In total, 435 participants (mean age: 38 years, 53.8% female) were included. The total Q-LES-Q-SF score indicated overall medium satisfaction with their quality of life (score of 44.3; possible range 14–70). The most prevalent side effects were “Sleepy during the day” (83.2%), “Difficulty sleeping” (74.7%), “Dry mouth” (63.9%), “Problems enjoying sex” (53.4%) and “Gaining weight” (52.4%). Women reported the side effects of “Sleepy during the day”, “Problems enjoying sex” and “Gaining weight” more frequently than men. Key side effects impacted physical, social, occupational and psychological aspects of functioning. Patients with key side effects often felt frustrated by their experiences. Total Q-LES-Q-SF score showed a significant inverse correlation with the score of pre-defined groups of side effects indicating worse QoL in association with more severe key side effects in these patients.

**Conclusion:**

Stable patients with schizophrenia taking second-generation antipsychotics live with many side effects, including activating and sedating side effects, sexual side effects, and weight gain. Presence of these side effects is associated with substantial impacts across all aspects of daily functioning and lower quality of life and satisfaction.

## Background

The second-generation antipsychotic medications used to treat schizophrenia and other psychiatric conditions were developed to have a lower propensity to cause the extrapyramidal side effects that significantly limit the utility of first-generation antipsychotics. However, while the tolerability profile of the second-generation agents is much improved, treatment is still associated with a variety of side effects [1, 2]. In one nationwide survey, 86.2% of patients reported at least one side effect due to their antipsychotic treatment [3].

Systematic review and meta-analyses have found that the different antipsychotics differ substantially in side effects [4]. Some of the most bothersome side effects of second-generation antipsychotics include those that can be grouped into *activating* (e.g., restlessness, feeling jittery, insomnia, and extrapyramidal symptoms), *sedating* (e.g., sleepiness, sedation, difficulty thinking or concentrating, and dizziness), and endocrine (e.g., sexual dysfunction, decreased interest in sex) and metabolic effects (e.g., weight gain), referred to as *other* side effects in this manuscript [3, 5, 6]. While the prevalence of these side effects with the various antipsychotic treatments are well documented [3, 4, 7–10], their impact on functioning and quality of life is under-researched and not as well understood from the patient perspective.

In addition, we still understand relatively little about how the patients themselves view their treatments and outcomes, and how this impacts their behavior. Such perspectives cannot be obtained from objective assessments of efficacy, side-effects, adherence, etc., and are important to understand if clinicians are to encourage patients to engage with treatment and actively manage their illness. By trying to understand the patient perspective, clinicians can work to ensure the acceptability of care provision [11]. The primary objective of the study was to understand how specific side effects impact daily functioning, emotional well-being, and overall quality of life (QoL) of patients with schizophrenia from their own perspective. A secondary objective was to investigate patients’ emotional responses to experiencing these side effects. The hypothesis was that patients with schizophrenia experiencing side effects from the use of second-generation antipsychotics are functionally impaired due to their side effects.

## Methods

### Recruitment and setting

This was a cross-sectional, web-based, self-reported survey. The study protocol was reviewed and approved by independent ethics review boards. All participants provided electronic informed consent and were provided with the study details prior to electing to participate.

Participants from the United States (US), Canada, Australia, Spain, Italy, Norway, and Denmark were recruited via market research agencies (Medpanel, Instar, and Global Perspectives) that utilized physicians, medical research patient panels and patient advocacy groups. Data collection procedures were in accordance with ethics standards ensuring patient confidentiality, anonymity throughout the study, and that patient-identifying information was not collected.

### Study sample

Eligible participants were adult patients (18- to 65-year old) who self-reported the following: a diagnosis of schizophrenia by a healthcare professional, being stable for ≥ 1 month at the time of screening (defined as having no inpatient hospitalizations, no visits to the emergency room, no suicide attempts, and remaining on the same medication regimen), taking the same second-generation antipsychotic for 1–12 months. Participants had to be experiencing at least one of the side effects included in the Glasgow Antipsychotic Side-Effect Scale (GASS)—a validated and licensed scale for use in this study [12]. To complete the web-based survey, participants were also required to have access to a computer. Each participant completed the survey in the local language. Participants taking first-generation antipsychotics as monotherapy or part of a multiple drug regimen were excluded.

### Survey design

A pilot questionnaire was developed by the authors and was tested with four participants with schizophrenia who completed the survey in the presence of a team member, and then answered usability and cognitive debriefing questions. The pilot study confirmed ease of use, clarity, relevance, and comprehension of the survey items and responses prior to launch of the main study survey.

The survey was developed in English, then translated and adapted for each country. It was designed to take approximately 20 min to complete. Patients first completed a short survey screening questionnaire, and if they fulfilled the inclusion and exclusion criteria they could go forward to the full survey. Survey questions included sociodemographic and clinical history as well as standardized assessments and questions related to the emotional impact of the side effects (Additional file 1). The standardized assessments embedded in the web survey were the GASS and the Quality of Life and Enjoyment Scale Short Form (Q-LES-Q-SF) [13]. The GASS is a self-reported questionnaire (22 items) designed to measure the frequency of recently experienced side effects due to antipsychotics. An additional item was added to the GASS for its clinical relevance (“Difficulty sleeping”), resulting in a total of 23 potential side effects. Item response options range from “Never” to “Every Day.” A total side effect burden score was derived by summing all the frequency scores of side effects reported. The Q-LES-Q-SF (16 items) assesses the degree of enjoyment and satisfaction experienced by patients in various areas of daily life and questions were linked to taking the current medication. The item response options range from “Very poor” to “Very good.” The total score ranges from 14 to 70; higher scores indicate better enjoyment and satisfaction with life.

To reduce participant burden, participants who indicated they experienced any of eight pre-defined ‘key’ side effects on the GASS then answered specifically developed questions related to the day-to-day functional and emotional impact of each side effect [including severity of impact rated on a 0–100 visual analog scale (VAS)]. These key side effects were chosen as potentially the most bothersome [3] and were categorized as *activating* (“Shaky hands or arms,” “Restlessness,” and “Difficulty sleeping”), *sedating* (“Sleepy during the day,” “Feeling drugged or like a zombie,” and “Feeling dizzy/Fainted”), and *other* side effects (“Problems enjoying sex” and “Gaining weight”). Sub-scores for each *activating*, *sedating*, and *other* side effect category were created by summing scores for the relevant side effects.

Since neither the GASS nor Q-LES-Q-SF consider functional or emotional impacts, the survey included questions specifically designed to explore these areas. Functional impact questions were tailored to the side effects and included items from 4 domains: physical (e.g., “Physical discomfort,” “Energy level,” and “Ability to concentrate”), social (e.g., “Ability to communicate with partner” and “Intimate relationships”), vocational (e.g., “Ability to get or do job”), and emotional (e.g., “Afraid to fall over” and “Fear of being rejected”). The emotional impact of experiencing side effects was further assessed using a list of descriptors including “Anguished,” “Apathetic/Indifferent,” “Ashamed/Embarrassed,” “Confused/Doubtful,” “Dissatisfied,” “Frustrated,” “Impatient/Irritated/Angry,” “Hopeless,” “Overwhelmed, Resigned,” and “Trusting/Accepting.”

### Analysis

All study data were analyzed using Statistical Analysis Software (SAS) version 9.4 (Cary, NC). Descriptive data are presented. Spearman correlations were analyzed for associations between GASS side effects and side effect categories, and with assessments of quality of life and satisfaction as measured on the Q-LES-Q-SF. Correlations were considered very weak to unrelated if *r*_s_ ≤ 0.19, weak between 0.20 and 0.35, moderate between 0.36 and 0.49, and strong if ≥ 0.5. Simple and multiple linear regression analyses were used to determine the best predictors of enjoyment and satisfaction of life as measured by the Q-LES-Q-SF. Subgroup analyses were conducted to test any sociodemographic characteristics which may have affected side-effect reporting and impact differences. Additional descriptive subgroup analyses were performed for age (dichotomous breakdown based on the median score of median age of 37-year old), sex (male/female) and employment status (employed = full- or part-time work or disabled and able to work, student, volunteer; unemployed = unemployed or disabled and unable to work; other = retired, or homemaker).

## Results

### Participant disposition and demographics

**Fig. 1:**
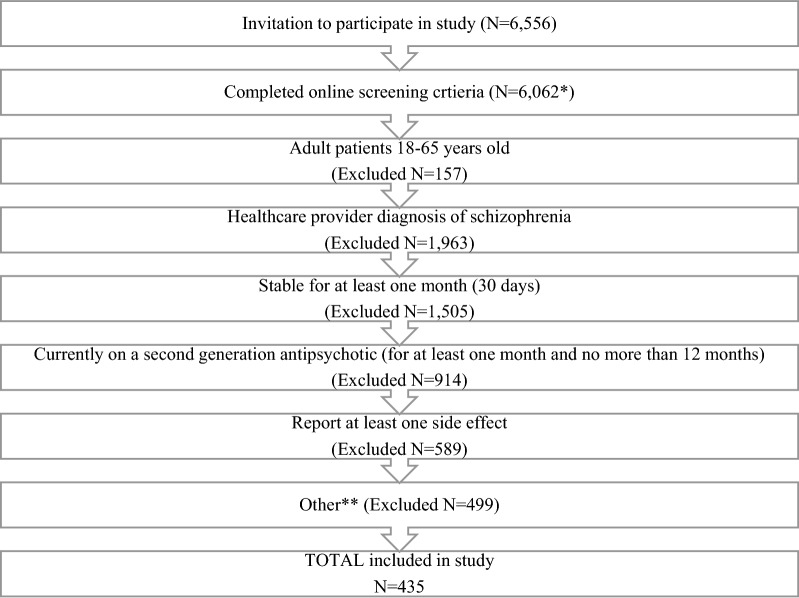
Screening Disposition for Participation in Online Patient Survey; including all participants from USA, Canada, Italy, Australia, Spain, Italy, Norway, and Denmark. * includes patients who started the screener but did not complete or continue, or those who attempted to take the survey more than once, or were removed due to duplicates. ** Other disqualifications included: not living in the relevant country, did not electronically consent, and/or duplicates

A total of 6556 patients were approached to participate in the study between September 2017 and October 2018. Of those, 6062 (91.1%) completed the online screening criteria and 435 (7.2%) met the final study inclusion/exclusion criteria and completed the full survey (see Fig. 1 for full details of screen failures). Overall, 53.8% of participants were women, and the average age was 37.9-year old (SD = 11.0; range 18–66). Most participants (72.0%) were diagnosed with schizophrenia within the last 5 years; 48.7% reported living with a spouse, partner, and/or child, while 20.5% lived with a parent/s and 16.1% lived alone. Sample characteristics for the overall study sample are provided in Table 1, and by country/region in Additional file 3: Table a.

**Table 1:** Sample summary (*n* = 435)

Variable		Variable	
Age (years)		Time since diagnosis of schizophrenia, *n* (%)	
Mean (SD) [Range]	37.9 (11.0) [18–66]	More than 10 years ago	48 (11.0)
Gender*, n* (%)		Between 5 and 10 years ago	74 (17.0)
Male	198 (45.5)	Between 1 and 5 years ago	210 (48.3)
Female	234 (53.8)	Less than 1 year ago	103 (23.7)
Employment status*, n* (%)*		Country*, n* (%)	
Employed, full-time or part-time	173 (39.8)	USA	180 (41.4)
Homemaker	65 (14.9)	Canada	99 (22.8)
Student	24 (5.5)	Australia	28 (6.4)
Volunteer	12 (2.8)	Italy	90 (20.7)
Unemployed	60 (13.8)	Spain	22 (5.1)
Retired	14 (3.2)	Denmark	8 (1.8)
Disabled and able to work	33 (7.6)	Norway	8 (1.8)
Disabled and unable to work	54 (12.4)	Living Situation*, n* (%)***	
Education*, n* (%)**		Spouse/partner and children	115 (26.4)
Elementary/primary school	21 (4.8)	Spouse/partner without children	97 (22.3)
Secondary/high school	123 (28.3)	Parent(s)	89 (20.5)
Some college/college degree	178 (40.9)	Alone	70 (16.1)
Some graduate school/graduate degree	76 (17.4)	Children without spouse/partner	19 (4.4)
Technical or vocational degree	23 (5.3)	Other (including family member)	14 (3.2)
Other	14 (3.2)	Group home, community facility	16 (3.7)

*SD* standard deviation

* Missing data: *n* = 58 (32.2%); ** Missing data: *n* = 5 (2.8%); *** Missing data: *n* = 1 (0.6%)

### Frequency of side effects

**Fig. 2:**
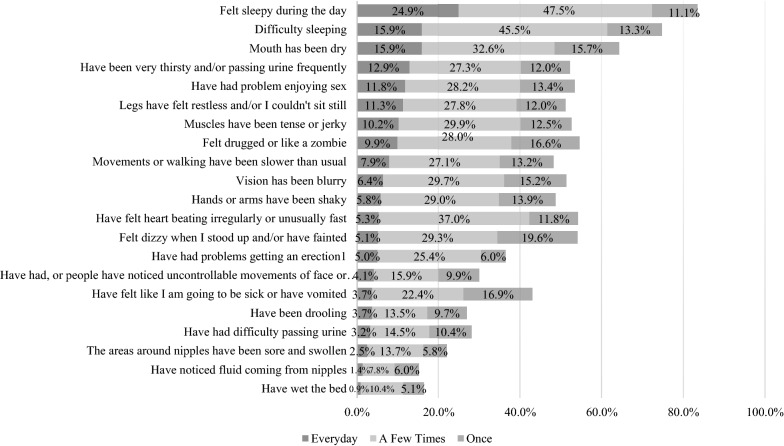
Frequency of GASS Side Effects (*n* = 435). Numeric answers to the questions range from 1–4, representing “Never” to “Everyday.” This figure does not report “Never” responses. “Change in periods” and “Gaining weight” are not reflected in this figure as the responses were categorical: Yes/No. There are a total of 23 items

The GASS total burden of side effects score was 18.5 (SD = 11.4), indicating moderate burden. The ‘top 3’ most frequently experienced side effects were “Sleepy during the day” (83.5%), “Difficulty sleeping” (74.7%), and “Mouth dry” (63.9%) (Fig. 2). Over half of all participants (52.4%) reported experiencing “Gaining weight.” Results were fairly similar across the countries (GASS burden scores were consistently in the moderate range and the top 3 side effects were the same) but some differences were apparent. For example, weight gain was most common in Italy and least common in the US (62.2% and 46.7%, respectively).

Descriptive subgroup analyses revealed that younger patients (< 37-year old) reported the key side effects of “Sleepy during the day”, “Drugged or Like a Zombie”, “Dizziness”, “Restlessness”, and “Difficulty Sleeping” more frequently than older people. Women also reported the side effects of “Sleepy during the day”, “Problems enjoying sex” and “Gaining weight” at a significantly greater frequency than men (Additional file 3: Tables b and c).

### Functional impact of side effects

**Fig. 3:**
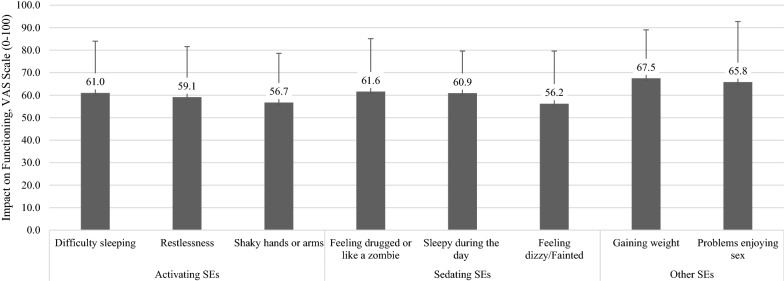
Impact of Side Effects on Functioning (*N* = 435). *VAS* Visual Analog Scale. Subset analysis based on whether key side effect is reported on the GASS scale. *Higher functional severity indicates worse impact

All key side effects were reported as having a moderate to severe overall impact on participants’ functioning (range 54.8 to 65.2) (defined by the VAS score ≥ 50), (Fig. 3). The two side effects with the most frequently reported impacts on functioning were both *sedating* side effects: “Feeling drugged or like a zombie” (75.1%) and “Sleepy during the day” (76.5%). Subgroup analyses showed some differences in reporting of the severity of impacts when categorized by employment status. There was at least a 5-point VAS difference between employed mean severity scores and unemployed mean severity scores for the key side effects, with scores higher (worse impact) for those unemployed (Additional file 3: Table d). All functional domains (including physical, social, vocational, and emotional) were impacted (Additional file 2). Across the activating and sedating key side effects, the most frequently reported functional impact (i.e., ranked first or second) was the “ability to get or do a job”.

### Emotional impact of side effects

*Activating and sedating* side effects led most often to reporting of being “Frustrated,” followed by feeling “Dissatisfied” (Table 2). For those participants who experienced “Problems enjoying sex” as a medication side effect, 37.2% reported feeling “Frustrated”; for those who experienced problems with “Gaining weight,” the majority reported feeling “Lack of confidence” (59.2%) as a result.

**Table 2:** Top three emotional descriptors reported by key side effect

Key side effect	Top three emotional descriptors	Representational comments from participants
Problems enjoying sex	Frustrated	“The lack of the positive things that come with satisfactory sexual relationships” “I no longer feel to be a man, I do not trust myself, I no longer have my virility and I feel frustrated, useless, done for as if my life had stopped, the ugliest thing is my resignation to this situation”
	Ashamed/embarrassed	
	Dissatisfied	
Gaining weight	Lack of confidence	“Basically, too big to move. I lose my breath walking from the front door to the letter box. I cannot stand up long enough to wash more than 2 dishes” “It is hard for me to walk, go upstairs, in my wardrobe I have clothes of all sizes because I still hope to lose weight someday”
	Ashamed/embarrassed	
	Frustrated	
Feeling drugged or like a zombie	Frustrated	“It makes me feel like a zombie, so talking to people is a real effort” “Feels like I have no energy to do things” “It’s difficult to perform everyday things like washing, cooking, etc.”
	Dissatisfied	
	Hopeless	
Sleepy during the day	Frustrated	“Going to visit friends who think that I am not interested because I appear to be asleep” “Just very low self-esteem. I hate the way that people think that I am being rude, disrespectful or not caring when I fall asleep. The fact that they think it is deliberate destroys me” “It affects me being able to get to classes on time”
	Dissatisfied	
	Impatient/irritated/angry	
Restlessness	Frustrated	“I feel like I need to do something important, but my family is content to stay at home watching their tv and computer screens” “It creates (gives) me anxiety and I cannot understand why, it scares me and prevents me from continuing to do any work”
	Impatient/irritated/angry	
	Dissatisfied	
Difficulty sleeping	Frustrated	“When I don’t sleep well, I am not as sharp the next day” “It is really alarming to wait to get to sleep, I cannot, despite taking drugs to sleep, because I do not get tired enough, I do not go out, I do nothing all day, I cannot read any more”
	Impatient/irritated/angry	
	Dissatisfied	
Shaky hands or arms	Frustrated	“It embarrasses me, so I don’t like meeting people or going to appointments” “To get dressed I take more time and also to do many other things”
	Ashamed/embarrassed	
	Dissatisfied	
Feeling dizzy/fainted	Frustrated	“Absolutely nothing that I can do well… Also my boss is bullying me because of my symptoms. I find it all overwhelming and unfair because doctors and psychologists don’t believe me when I tell them that I think my symptoms are related to the medication” “I sit down, and I wait that they pass off” “I have difficulties to plan the jobs to do because my head is spinning, and I don’t feel safe”
	Confused/doubtful Overwhelmed	

Based on highest percent reporting the descriptor, and only asked to those who experienced that key side effect. Items were pre-selected and participant was able to select as many responses as they wish

### Quality of Life Satisfaction

**Fig. 4:**
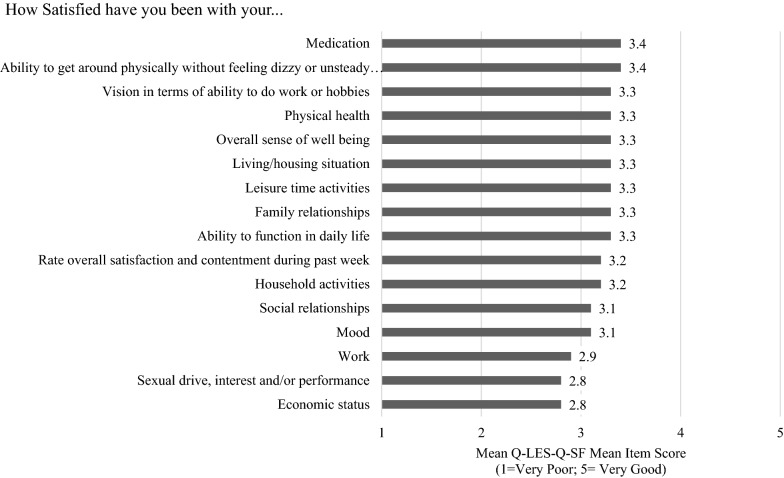
Quality of Life and Satisfaction Short Form (Q-LES-Q-SF) Scores-(*N* = 435). Subset analysis based on whether key side effect is reported on the GASS scale

Despite a high incidence of side effects, the total score on the Q-LES-Q-SF was 44.3 (SD: 9.8), indicating medium overall satisfaction with quality of life (Fig. 4). Regression analyses showed that age and sex were also relevant for the impact of activating side-effects on quality of life and sex was also relevant for the impact of sedating side-effects on quality of life (Additional file 3: Tables e–g). Subgroup comparisons found that women reported a worse quality of life total score compared to men (43.0 vs 45.8, *p* < 0.0005), and those who were unemployed fared worse compared to those employed or other (e.g., retired or homemaker) (40.9 vs. 46.3 and 43.0, respectively, *p* < 0.0001) (Additional file 3: Tables h and i). “Sexual drive, interest and/or performance,” “Economic status,” and “Work” had the lowest Q-LES-Q-SF scores (< 3), indicating a less than “fair” level of satisfaction. The highest Q-LES-Q-SF scores were observed for satisfaction with “Medication” and the “Ability to get around physically without feeling dizzy” (mean score of 3.4), indicating a fair satisfaction level.

### Correlational analyses

Weak to moderate but statistically significant inverse correlations between side effects and the total Q-LES-Q-SF were seen for all key side effects confirming the negative impact of side effects on life satisfaction. Significant correlations were observed between the total Q-LES-Q-SF score and total GASS score (*r* = − 0.34, *p* < 0.0001), as well as the individual side effects “Feeling drugged or like a zombie” (*r* = − 0.29, *p* < 0.0001), “Difficulty sleeping” (*r* = − 0.26, *p* < 0.0001), and “Problems enjoying sex” (*r* = − 0.26, *p* < 0.0001) (Table 3).

**Table 3:** Association between the Key Side Effects and Total GASS Score with the Q-LES-Q-SF Total Score

Key side effects and total GASS Score	Side effect category	*N*	Total Q-LES-Q-SF Score Spearman correlation coefficients
Sleepy during the day	Sedating	434	− 0.23**
Feeling drugged or like a zombie	Sedating	435	− 0.29**
Feeling dizzy/fainted	Sedating	433	− 0.22**
Difficulty sleeping	Activating	435	− 0.26**
Shaky hands or arms	Activating	431	− 0.19**
Restlessness	Activating	432	− 0.25**
Problems enjoying sex	Other	432	− 0.26**
Gaining weight	Other	427	− 0.09*
Total GASS Score	NA	435	− 0.34**

*GASS* Glasgow Antipsychotic Side-Effect Scale, *Q-LES-Q-SF* Quality of Life Enjoyment and Satisfaction Questionnaire Short Form

*Denotes *p* value significant at *p* < 0.05 and for **p < 0.001

### Regression

Table 4 presents a multivariate regression using the GASS categories as predictors (*sedating*, *activating*, and *other* side effects) adjusting for demographic variables on quality of life satisfaction, as measured by the Q-LES-Q-SF total score. The multivariate model demonstrated that the presence of sedating side effects, other side effects, and age were significant predictors of poor life satisfaction within the model (statistical significance of at least 0.05 in the model). In other words, older age (− 0.18, SE = 0.06), a greater frequency of *sedating* (− 3.52, SE = 0.94), and *other* side effects (− 1.73, SE = 0.75) significantly predicted lower enjoyment and satisfaction with life, whereas activating side effects were not significantly associated with poor quality of life in this model.

**Table 4:** Linear regression model of HRQoL on GASS categories and demographic characteristics

Predictor: side effect GASS categories	Total Q-LES-Q-SF Score (*N* = 435) Multivariate estimate (SE)
Activating side effects	− 0.87 (0.86)
Sedating side effects	− 3.52 (0.94)*
Other side effects	− 1.73 (0.75)*
Age (continuous)	− 0.18 (0.06)*
Gender: female	− 1.68 (1.21)
Race/ethnicity: white	0.06 (1.80)
Education: at least some college	0.08 (1.21)
Employment status: employed (full or part time)	1.29 (1.16)
Living situation: live alone	− 0.40 (1.44)
Time since diagnosis (years, continuous)	0.31 (0.65)

*SE* Standard Error, *GASS* Glasgow Antipsychotic Side-Effect Scale; *HRQoL* health-related quality of life, *Q-LES-Q-SF* Quality of Life Enjoyment and Satisfaction Questionnaire-Short Form. *Activating* side effects refer to restlessness, shaky hands or arms, and difficulty sleeping; *sedating* refers to sleepiness, feeling drugged or like a zombie and dizziness. *Other* refers to weight gain and problems enjoying sex. *p value significant at *p* < 0.05

## Discussion

To our knowledge, this is one of the first surveys to provide in-depth evaluation of the functional and emotional impacts and quality of life associated with antipsychotic side effects from the patient perspective. The results of this patient-reported survey confirm the burden of side effects of second-generation antipsychotic medications and also show how side effects impact daily functioning and quality of life satisfaction.

The survey was designed to understand the impact of key side effects on daily life at a granular level; however, we found that if the side effect is present, all aspects of functioning are affected. For example, while sedation (sleepy during the day) was most strongly associated with the vocational aspect “Ability to get a job or do your job”, it also impacted social (“Afraid to go out”) and physical (“Energy level”) domains.” Although functional impact was investigated using tailored questioning (according to the relevance of each side effect), there were some clear patterns across all side effects. In particular, 6 of the 8 key side effects were rated by the participants as having a high impact on their “Ability to get or keep a job”. The high impact of side effects on work was further supported by the Q-LES-Q-SF scores for which “Work” had the lowest scores (< 3), indicating a less than “fair” level of satisfaction. Participants also frequently cited work impacts when discussing their side effects (in open text questions). For example, when discussing the impact of restlessness one participant noted *“It [restlessness] creates anxiety and I cannot understand why, it scares me and prevents me from continuing to do any work.”* Another participant discussing how they felt sleepy during the day noted that it affected their *“Being able to get to classes on time”.* While causality cannot be implied, it is also pertinent that mean side effect severity scores were consistently (at least 5 points) higher in unemployed participants compared with those in employment.

We found that women reported the side effects of “Gaining weight”, “Problems enjoying sex” and “Sleepy during the day”, more frequently than men. This is in line with systematic reviews which have found that women have an increased susceptibility to weight gain and specific cardiovascular risks of antipsychotics [14, 15]. However, while other reviews have found higher rates of hyperprolactinaemia in women, they have found that sexual dysfunction is reportedly more common in men than women (50% in men vs. 25–50% in women) [16]. The discrepancies in our findings may reflect the targeted questioning rather than reporting as an adverse event in a clinical study. Nevertheless, our data highlight the need to better understand the differential impact of side effects in women and men, especially given that a recent naturalistic study of 1087 patients with psychosis found that twice as many women as men described their side effect burden as severe [17].

Only a few studies have confirmed that side effects have an impact beyond the physical experience of the side effect, and also impact quality of life and emotional functioning [18, 19]. A key strength of our research methodology is that we asked for direct input from people living with schizophrenia (self-reported) rather than relying on information from clinicians or caregivers. At the same time, it is worth noting the significant recruitment challenges associated with recruiting people with stable schizophrenia [20, 21]. We screened 6556 patients to obtain a final sample of 435 participants (6.7% of the total patients screened). Most participants were screened out for either not meeting stability criteria or not being diagnosed by an HCP; few participants were actually screened out for not experiencing side effects. By screening for people with stable schizophrenia, we may have skewed the population toward a more functional sample than in other schizophrenia studies. While this was not our intent, the sample likely does reflect a subpopulation of people with schizophrenia for whom the impacts of side effects on daily function are particularly important.

Correlational analyses in the current study indicated that all key side effects were weakly (0.09–0.34) but significantly associated with quality of life and life satisfaction. Side effects were also reported to have emotional and psychological consequences. Feeling “frustrated,” followed by feeling “dissatisfied,” was the topmost frequently reported emotional consequences for the eight side effects. We employed regression models to evaluate whether side effects had an independent effect on quality of life and life satisfaction, even when controlling for other factors. An important finding was that both the presence of *sedating* and *other* side effects (“Problems enjoying sex” and “Gaining weight”), along with age, were significant predictors of quality of life and life satisfaction even when accounting for activating side effects, sex, ethnicity, education, employment status, living situation, and time since diagnosis. There was a negative association between quality of life and impacts on functioning due to the side effects, as would be expected. Interestingly (and perhaps counter-intuitively), *activating* side effects were not significantly associated with quality of life and life satisfaction to a statistically significant extent.

## Limitations

Although the survey results provide new insights into the patient perspective on the impact on physical and emotional functioning of side effects associated with the use of second-generation antipsychotics, the study had several limitations. Key limitations include those inherent with all patient surveys, including reliance on patient self-report rather than clinician verification, and recall bias. In the absence of clinical information, we were unable to categorize participants in any way (e.g., by severity, predominant symptoms, etc.). To minimize survey burden, we did not collect data on factors such as age at onset of schizophrenia, other medical comorbidities and pharmacological treatments. In addition, it seems highly likely that the recruitment strategy skewed the sample toward a younger, higher functioning subpopulation of patients with schizophrenia. Most of the sample was diagnosed within the past 5 years, had received some formal education, and were in relationships. These individuals might be the most affected by physical and emotional functional impacts of side effects, as they may have higher functioning than more severely affected chronic patients.

To simplify the survey, we focused on side effects that had been described in the literature to be the most bothersome to patients with schizophrenia [3, 22], and further work should investigate whether other side effects impact all aspects of life in the same way. While the GASS and Q-LES-Q-SF are validated instruments, the questions related to functional and emotional impacts were specifically designed for this survey. At this stage, it was not our intent to compare impacts between the various second-generation antipsychotics. Future work should be carried out with relevant sample sizes for each drug, and doses will be important to capture.

It is also relevant that participants answered the web survey in a comfortable environment, such as at home or at a library in their community. It could be argued that participants in this setting may have been more comfortable divulging sensitive issues such as sex, weight gain, and other potentially uncomfortable subjects. Finally, it is important to emphasize that functioning in schizophrenia is multifactorial, including many domains not related to side effects including symptom severity, health status, social cognition and environmental factors. However, side effects may be one of the few factors that can be changed/modified to improve a person’s chances for functional recovery.

## Conclusion

This survey highlights the importance of capturing the patients’ perspective when evaluating the impacts of second-generation antipsychotics. Our findings confirm that stable patients with schizophrenia taking second-generation antipsychotics are living with many side effects, including activating and sedating side effects, sexual side effects, and weight gain. Activating, sedating as well as metabolic and sexual side effects are associated with substantial impacts across all aspects of daily functioning (physical, social, vocational and emotional), as well as lower quality of life satisfaction. These effects were present even when controlling for several sociodemographic and disease-related factors. Further research is warranted to further evaluate the associations between side effects and functional impacts, how they interact with schizophrenia symptoms, and how the functional impacts can be mitigated.

## Supplementary information

**Additional file 1.** Impact of Side effects on functioning and emotion Survey Items (programmed via a web survey).

**Additional file 2.** Impact of Side Effects on Functioning.

**Additional file 3. Table a**. Sample characteristics by region. **Table b**. Mean Side Effect Scores as measured by the Glasgow Antipsychotic Side-Effect Scale (GASS), All Countries Combined by Age and Overall. **Table c**. Mean Side Effect Scores as measured by the Glasgow Antipsychotic Side-Effect Scale (GASS), All Countries Combined by Gender and Overall. **Table d**. Mean Severity (VAS Scales) of Key Side Effects’ Impact on Functioning (Subset analysis), All Countries Combined by Employment Status and Overall. **Table e**. Linear Regression Model of Activating Side Effects, Demographics, and Time Since Diagnosis on HRQoL (Model A). **Table f**. Linear Regression Model of Sedating Side Effects, Demographics, and Time Since Diagnosis on HRQoL (Model B). **Table g.** Linear Regression Linear Regression Model of Other Side Effects, Demographics, and Time Since Diagnosis on HRQoL (Model C). **Table h.** Quality of Life Enjoyment and Satisfaction Questionnaire Short Form (Q-LES-Q-SF) Item Descriptive Statistics, All Countries Combined by Gender and Overall. **Table i.** Quality of Life Enjoyment and Satisfaction Questionnaire Short Form (Q-LES-Q-SF) Item Descriptive Statistics, All Countries Combined by Employment Status and Overall

## Data Availability

The datasets supporting the conclusions of this article are available from the corresponding author upon reasonable request and the survey materials have been added to the additional file.

## Abbreviations

GASS: Glasgow Antipsychotic Side-Effect Scale
HRQoL: Health-related quality of life
Q-LES-Q-SF: Quality of Life Enjoyment and Satisfaction Questionnaire Short Form
SAS: Statistical Analysis Software
SD: Standard deviation
SE: Standard error
US: United States
VAS: Visual analog scale
