# Correction to: The impact on functioning of second-generation antipsychotic medication side effects for patients with schizophrenia: a worldwide, cross-sectional, web-based survey

**DOI:** 10.1186/s12991-020-00294-3

**Published:** 2020-08-10

**Authors:** Rajiv Tandon, William R. Lenderking, Catherine Weiss, Huda Shalhoub, Carla Dias Barbosa, Jun Chen, Mallik Greene, Stine R. Meehan, Laëtitia Bouérat Duvold, Celso Arango, Ofer Agid, David Castle

**Affiliations:** 1 Western Michigan University Homer Stryker M.D. School of Medicine, 300 Portage Street, Kalamazoo, MI 49007 USA; 2 Evidera, 7101 Wisconsin Avenue, Suite 1400, Bethesda, MD 20814 USA; 3 Otsuka Pharmaceutical Development & Commercialization, Inc., 508 Carnegie Center Drive, Princeton, NJ 08540 USA; 4 H. Lundbeck A/S, Ottiliavej 9, 2500 Valby, Denmark; 5 Department of Child and Adolescent Psychiatry, Institute of Psychiatry and Mental Health, Hospital General Universitario Gregorio Marañón, IiSGM,CIBERSAM, School of Medicine, Universidad Complutense, Calle del Dr. Esquerdo, 46, 28007 Madrid, Spain; 6 Schizophrenia Division, Complex Care & Recovery Program, Centre for Addiction and Mental Health, Toronto, Canada; 7 Department of Psychiatry and Institute of Medical Science, University of Toronto, 1001 Queen Street West, Toronto, ON M6J 1H4 Canada; 8 St Vincent’s Health and The University of Melbourne, 41 Victoria Parade, Fitzroy, VIC 3065 Australia

## Correction to: Ann Gen Psychiatry (2020) 19:42 10.1186/s12991-020-00292-5

Following publication of the original article [1], the authors identified an error in Fig. 3. The correct Fig. 3 is given below.

**Fig. 3:**
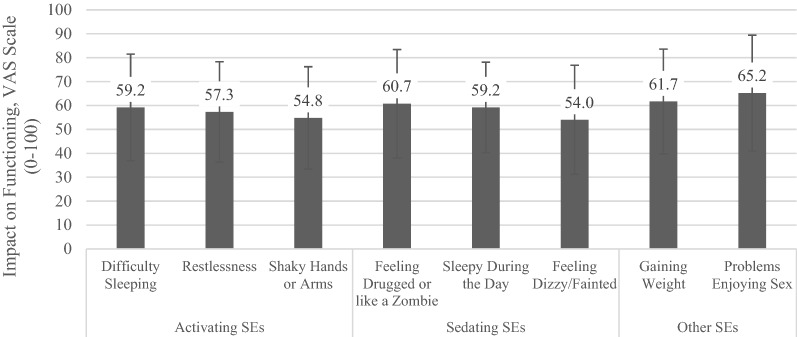
Impact of Side Effects on Functioning (*N* = 435). VAS Visual Analog Scale. Subset analysis based on whether key side effect is reported on the GASS scale. *Higher functional severity indicates worse impact

## References

[1] Tandon R, Lenderking WR, Weiss C, Shalhoub H, Barbosa CD, Chen J, Greene M, Meehan SR, Duvold LB, Arango C, Agid O, Castle D (2020). The impact on functioning of second-generation antipsychotic medication side effects for patients with schizophrenia: a worldwide, cross-sectional, web-based survey. Ann Gen Psychiatry.

