# Supplementary material for: The impact on functioning of second-generation antipsychotic medication side effects for patients with schizophrenia: a worldwide, cross-sectional, web-based survey
Source: Ann Gen Psychiatry. 2020 Jul 13;19:42. doi: 10.1186/s12991-020-00292-5 (PMC7359579; doi:10.1186/s12991-020-00292-5)
Supplement: Supplementary file 1 — Additional file 1. Impact of Side effects on functioning and emotion Survey Items (programmed via a web survey). [file 12991_2020_292_MOESM1_ESM.pdf]

ADDITIONAL FILE 1

Impact of Side effects on functioning and emotion Survey Items (programmed via a web survey)

A. SLEEPINESS

[SKIP IF "NEVER" SELECTED FOR ITEM 1 OF GASS]

1.You have said that since taking your current medication, you have **felt sleepy during the day**. When did you last experience the feeling of being **sleepy during the day**?

O_1 In the past day

O_2 In the past week

O_3 In the past month

O_4 In the past 3 months

O_5 In the past year

<PAGE BREAK>

2. Has this **feeling of sleepiness during the day** been affecting your <u>energy level</u>?

O_1 Yes O_2 No

[SHOW IF YES] Please indicate on the scale; how much has **sleepiness during the day affected** your <u>energy level</u>? *(To enter your response below, click on the circle and drag it to the left or right to show how much your energy level was affected)*

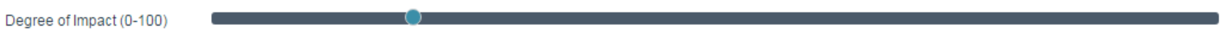

<PAGE BREAK>

3. Has this **feeling of sleepiness during the day** been affecting your <u>ability to do chores around the house</u>?

O_1 Yes O_2 No

[SHOW IF YES] Please indicate on the scale; how much has **sleepiness during the day** affected your ability to do chores around the house? *(To enter your response below, click on the circle and drag it to the left or right to show how much your ability to do chores around the house was affected)*

Degree of Impact (0-100)

<PAGE BREAK>

4. Has this **feeling of sleepiness during the day** been affecting your ability to take care of yourself?

O$_1$ Yes O$_2$ No

[SHOW IF YES] Please indicate on the scale; how much has **sleepiness during the day** affected your ability to take care of yourself?

Degree of Impact (0-100)

<PAGE BREAK>

5. Has this **feeling of sleepiness during the day** been affecting you avoiding/ not talking to others?

O$_1$ Yes O$_2$ No

[SHOW IF YES] Please indicate on the scale; **how much has sleepiness during the day** affected how much you avoid/not talk to others?

Degree of Impact (0-100)

<PAGE BREAK>

6. Has this **feeling of sleepiness during the day** made you afraid to go out?

O$_1$ Yes O$_2$ No

[SHOW IF YES] Please indicate on the scale; **how much has sleepiness during the day** affected how afraid you are to go out?

Degree of Impact (0-100)

<PAGE BREAK>

7. Has this **feeling of sleepiness during the day** made you not be able to get a job or do your job?

O$_1$ Yes O$_2$ No

[SHOW IF YES] Please indicate on the scale; **how much has sleepiness during the day** affected your ability to get a job or do your job?

Degree of Impact (0-100)

<PAGE BREAK>

8. Has this **feeling of sleepiness during the day** been affecting any other aspects of your daily functioning?

O$_1$ Yes O$_2$ No

[SHOW IF YES] Please describe the other aspect(s):

____

[If Yes] Please indicate on the scale; **how much has sleepiness during the day** affected other aspects of your daily functioning?

Degree of Impact (0-100)

<PAGE BREAK>

9. **Sleepiness during the day** has an impact on my functioning. ☐$_1$ True ☐$_2$ False.

<PAGE BREAK>

10. How has this **feeling sleepiness during the day** made you feel? (Please check all that apply – if any)

- ☐ Apathetic/Indifferent
- ☐ Frustrated
- ☐ Hopeless
- ☐ Dissatisfied
- ☐ Ashamed/Embarrassed
- ☐ Trusting/Accepting
- ☐ Overwhelmed
- ☐ Confused/Doubtful
- ☐ Impatient/Irritated/Angry
- ☐ Resigned
- ☐ None of the above **[EXCLUSIVE]**

<PAGE BREAK>

B. RESTLESSNESS

[SKIP IF "NEVER" SELECTED FOR ITEM 7 OF GASS]

1. You have said that since taking your current medication you have felt restless and you could not sit still. How long has it been since you experienced **feeling restless and unable to sit still**?

O$_1$ In the past day

O$_2$ In the past week

O$_3$ In the past month

O$_4$ In the past 3 months

O$_5$ In the past year

<PAGE BREAK>

2. Has **feeling restless and unable to sit still** been affecting your energy level?

O$_1$ Yes O$_2$ No

[SHOW IF YES] Please indicate on the scale; **how much has feeling restless and not being able to sit still** affected your energy level? *(to enter your response below, click on the circle and drag your mouse to the left or right)*

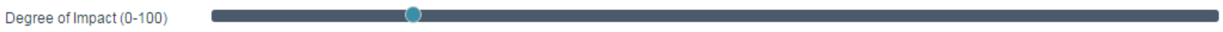

<PAGE BREAK>

3. Has **feeling restless and unable to sit still** been affecting your level of physical discomfort?

O$_1$ Yes O$_2$ No

[SHOW IF YES] Please indicate on the scale; **how much has feeling restless and not being able to sit still** affected your level of physical discomfort? *(to enter your response below, click on the circle and drag your mouse to the left or right)*

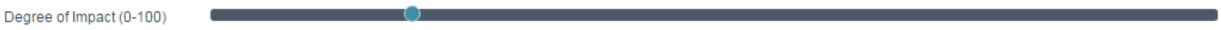

<PAGE BREAK>

4. Has **feeling restless and unable to sit still** been affecting your ability to do chores around the house?

O$_1$ Yes O$_2$ No

[SHOW IF YES] Please indicate on the scale; **how much has feeling restless and unable to sit still** affected your ability to do chores around the house?

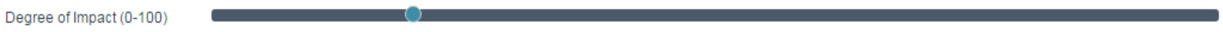

<PAGE BREAK>

5. Has **feeling restless and unable to sit still** been affecting your ability to take care of yourself?

O_1 Yes O_2 No

[SHOW IF YES] Please indicate on the scale; **how much has feeling restless and unable to sit still** affected your ability to take care of yourself?

O_1 Very little

O_2 A little

O_3 Moderately

O_4 Very Much

O_5 Severely

<PAGE BREAK>

6. Has this **feeling restless and unable to sit still** been affecting avoiding or not talking to others?

O_1 Yes O_2 No

[SHOW IF YES] Please indicate on the scale, **how much has feeling restless and unable to sit still** affected how much you avoid or not talk to others?

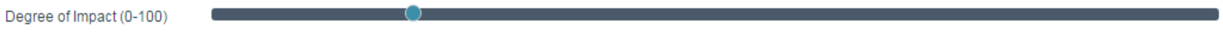

<PAGE BREAK>

7. Has **feeling restless and unable to sit still** been making you afraid to going out?

O_1 Yes O_2 No

[SHOW IF YES] Please indicate on the scale; **how much has feeling restless and unable to sit still** affected how afraid you are to go out?

Degree of Impact (0-100)

<PAGE BREAK>

8. Has **feeling restless and unable to sit still** been affecting your ability to get a job or do your job?

O_1 Yes O_2 No

[SHOW IF YES] Please indicate on the scale; **how much has feeling restless and unable to sit** still affected your ability to get a job or do your job?

Degree of Impact (0-100)

<PAGE BREAK>

9. Has **feeling restless and unable to sit still** been affecting any other aspects of your daily functioning?

O_1 Yes O_2 No

[SHOW IF YES Please describe the other aspect(s):

____

[SHOW IF YES] Please indicate on the scale; **how much has feeling restless and unable to sit** still affected other aspects of your daily functioning?

Degree of Impact (0-100)

<PAGE BREAK>

10. **Feeling restless and unable to sit still** has an impact on my functioning. ☐$_1$ True ☐$_2$ False

<PAGE BREAK>

11. How has **feeling restless or unable to sit still** made you feel? (Please check <u>all</u> that apply – if any)

- ☐ Apathetic/Indifferent
- ☐ Frustrated
- ☐ Hopeless
- ☐ Dissatisfied
- ☐ Ashamed/Embarrassed
- ☐ Trusting/Accepting
- ☐ Overwhelmed
- ☐ Confused/Doubtful
- ☐ Impatient/Irritated/Angry
- ☐ Resigned
- ☐ Anguished
- ☐ None of the above **[EXCLUSIVE]**

<PAGE BREAK>

C. TREMOR

[SKIP IF "NEVER" SELECTED FOR ITEM 6 OF GASS]

1. You have said that since taking your current medication, **your hands or arms have been shaky (tremor).** How long has it been since you experienced **your hands or arms being shaky (tremor)?**

O_1 In the past day

O_2 In the past week

O_3 In the past month

O_4 In the past 3 months

O_5 In the past year

<PAGE BREAK>

2. Has this **tremor** been affecting your energy level?

O_1 Yes O_2 No

[SHOW IF YES] Please indicate on the scale; **how much has having a tremor affected your energy level?** *(to enter your response below, click on the circle and drag your mouse to the left or right)*

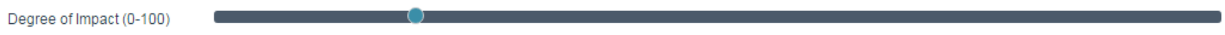

<PAGE BREAK>

3. Has this **tremor** been affecting your ability to do chores around the house?

O_1 Yes O_2 No

[SHOW IF YES] Please indicate on the scale; how much has having a **tremor** affected your ability to do chores around the house? *(to enter your response below, click on the circle and drag your mouse to the left or right)*

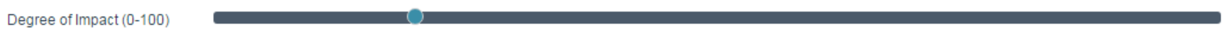

<PAGE BREAK>

4. Has this **tremor** been affecting your ability to take care of yourself?

O_1 Yes O_2 No

[SHOW IF YES] Please indicate on the scale; how much has having a **tremor** affected your ability to take care of yourself?

Degree of Impact (0-100)

<PAGE BREAK>

5. Has this **tremor** been affecting how much you avoid or not talk to others?

O_1 Yes O_2 No

[SHOW IF YES] Please indicate on the scale; Please indicate on the scale; how much has having a **tremor** affected how much you avoid others?

Degree of Impact (0-100)

<PAGE BREAK>

6. Has this **tremor** been affecting how much you are afraid to go out?

O_1 Yes O_2 No

[SHOW IF YES] Please indicate on the scale; Please indicate on the scale; how much has having a **tremor** affected how afraid you are to go out?

Degree of Impact (0-100)

<PAGE BREAK>

7. Has this **tremor** been affecting your ability to get a job or do your job?

O_1 Yes O_2 No

[SHOW IF YES] Please indicate on the scale; Please indicate on the scale; how much has having a **tremor** affected your ability to get a job or do your job?

Degree of Impact (0-100)

<PAGE BREAK>

8. Has this **tremor** been affecting your ability to write?

O_1 Yes O_2 No

[SHOW IF YES] Please indicate on the scale; Please indicate on the scale; how much has having a **tremor** affected your <u>ability to write</u>?

Degree of Impact (0-100)

<PAGE BREAK>

9. Has this **tremor** been affecting your <u>ability to grab a glass</u>?

O_1 Yes O_2 No

[SHOW IF YES] Please indicate on the scale; Please indicate on the scale;how much has having a **tremor** affected your ability to <u>grab a glass</u>?

Degree of Impact (0-100)

<PAGE BREAK>

10. Has this **tremor** been affecting <u>any other aspect of your daily functioning</u>?

O_1 Yes O_2 No

[SHOW IF YES] Please describe the other aspect(s):

____

[SHOW IF YES] Please indicate on the scale; how much has having a **tremor** affected <u>other aspects of your daily functioning</u>?

Degree of Impact (0-100)

<PAGE BREAK>

11. **Tremor** has an impact on my functioning. $\square_1$ True $\square_2$ False

<PAGE BREAK>

12. How has your **tremor** made you feel? (Please check <u>all</u> that apply – if any)

- ☐ Apathetic/Indifferent
- ☐ Frustrated
- ☐ Hopeless
- ☐ Dissatisfied
- ☐ Ashamed/Embarrassed
- ☐ Trusting/Accepting
- ☐ Overwhelmed
- ☐ Confused/Doubtful
- ☐ Impatient/Irritated/Angry
- ☐ Resigned
- ☐ None of the above **[EXCLUSIVE]**

<PAGE BREAK>

D. FEELING DRUGGED/LIKE A ZOMBIE

[SKIP IF “NEVER” SELECTED FOR ITEM 2 OF GASS]

1. You have said that since taking your current medication that you **felt drugged or like a zombie**. How long has it been since you experienced **feeling drugged or like a zombie**?

O1 In the past day

O2 In the past week

O3 In the past month

O4 In the past 3 months

O5 In the past year

<PAGE BREAK>

2. Has **feeling drugged or like a zombie** been affecting <u>your energy level</u>?

O1 Yes O2 No

[SHOW IF YES] Please indicate on the scale; how much has feeling drugged or like a zombie affected your <u>energy level</u>? *(to enter your response below, click on the circle and drag your mouse to the left or right)*

Degree of Impact (0-100)

<PAGE BREAK>

3. Has **feeling drugged or like a zombie** been affecting your <u>ability to do chores around the house</u>?

O_1 Yes O_2 No

[SHOW IF YES] Please indicate on the scale; how much has feeling drugged or like a zombie affected your <u>ability to do chores around the house</u>? *(to enter your response below, click on the circle and drag your mouse to the left or right)*

Degree of Impact (0-100)

<PAGE BREAK>

4. Has this **feeling drugged or like a zombie** been affecting your <u>ability to take care of yourself</u>?

O_1 Yes O_2 No

[SHOW IF YES] Please indicate on the scale; how much has feeling drugged or like a zombie affected your <u>ability to take care of yourself</u>?

Degree of Impact (0-100)

<PAGE BREAK>

5. Has **feeling drugged or like a zombie** been affecting avoiding or not talking to others?

O_1 Yes O_2 No

[SHOW IF YES] Please indicate on the scale; how much has feeling drugged or like a zombie affected how much you avoid or not talk to others?

Degree of Impact (0-100)

<PAGE BREAK>

6. Has **feeling drugged or like a zombie** been making you afraid to go out?

O_1 Yes O_2 No

[SHOW IF YES] Please indicate on the scale; how much has feeling drugged or like a zombie affected how afraid you are to go out?

Degree of Impact (0-100)

<PAGE BREAK>

7. Has **feeling drugged or like a zombie** been affecting your ability to concentrate?

O_1 Yes O_2 No

[SHOW IF YES] Please indicate on the scale; how much has feeling drugged or like a zombie affected your ability to concentrate?

Degree of Impact (0-100)

<PAGE BREAK>

8. Has **feeling drugged or like a zombie** been affecting your memory?

O_1 Yes O_2 No

[SHOW IF YES] Please indicate on the scale; how much has feeling drugged or like a zombie affected your memory?

Degree of Impact (0-100)

<PAGE BREAK>

9. Has **feeling drugged or like a zombie** been affecting your ability to get a job or do your job?

O$_1$ Yes O$_2$ No

[SHOW IF YES] Please indicate on the scale; how much has feeling drugged or like a zombie affected your ability to get a job or do your job?

Degree of Impact (0-100)

<PAGE BREAK>

10. Has this **feeling drugged or like a zombie** been affecting any other aspects of your daily functioning?

O$_1$ Yes O$_2$ No

[SHOW IF YES] Please describe the other aspect(s):

____

[SHOW IF YES] Please indicate on the scale; how much has feeling restless and not being able to sit still affected other aspects of your daily functioning?

Degree of Impact (0-100)

<PAGE BREAK>

11. **Feeling drugged or like a zombie** has an impact on my functioning. ☐$_1$ True ☐$_2$ False

<PAGE BREAK>

12. How does **feeling drugged or like a zombie** make you feel? (Please check <u>all</u> that apply – if any)

- ☐ Apathetic/Indifferent
- ☐ Frustrated
- ☐ Hopeless
- ☐ Dissatisfied
- ☐ Ashamed/Embarrassed
- ☐ Trusting/Accepting
- ☐ Overwhelmed
- ☐ Confused/Doubtful
- ☐ Impatient/Irritated/Angry
- ☐ Resigned
- ☐ None of the above **[EXCLUSIVE]**

<PAGE BREAK>

E. DIZZINESS

[SKIP IF "NEVER" SELECTED FOR ITEM 3 OF GASS]

1. You have said that since taking your current medication you have **felt dizzy when you stood up.** How long has it been since you experienced **feeling dizzy when you stood up**?

O1 In the past day

O2 In the past week

O3 In the past month

O4 In the past 3 months

O5 In the past year

<PAGE BREAK>

2. Has your **feeling dizzy** been affecting your <u>energy level</u>?

O_1 Yes O_2 No

[SHOW IF YES] Please indicate on the scale; how much has feeling dizzy affected your <u>energy level</u>? *(to enter your response below, click on the circle and drag your mouse to the left or right)*

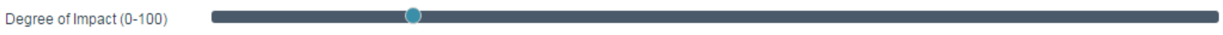

<PAGE BREAK>

3. Has **feeling dizzy** been affecting your <u>level of physical discomfort</u>?

O_1 Yes O_2 No

[SHOW IF YES] Please indicate on the scale; how much has feeling dizzy affected your <u>level of physical discomfort</u>? *(to enter your response below, click on the circle and drag your mouse to the left or right)*

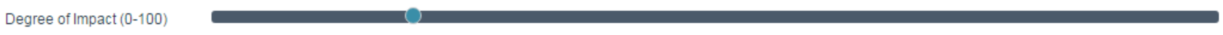

<PAGE BREAK>

4. Has **feeling dizzy** been affecting your <u>ability to do chores around the house</u>?

O_1 Yes O_2 No

[SHOW IF YES] Please indicate on the scale; how much has feeling dizzy affected your <u>ability to do chores around the house</u>?

Degree of Impact (0-100)

<PAGE BREAK>

5. Has **feeling dizzy** been affecting your ability to take care of yourself?

O_1 Yes O_2 No

[SHOW IF YES] Please indicate on the scale; how much has feeling dizzy affected your ability to take care of yourself?

Degree of Impact (0-100)

<PAGE BREAK>

6. Has **feeling dizzy** been affecting avoiding or not talking to others?

O_1 Yes O_2 No

[SHOW IF YES] Please indicate on the scale; how much has feeling dizzy affected how much you avoid or not talk to others?

Degree of Impact (0-100)

<PAGE BREAK>

7. Has **feeling dizzy** been affecting affected how afraid you are of falling over?

O_1 Yes O_2 No

[SHOW IF YES] Please indicate on the scale; how much has feeling dizzy affected how afraid you are of falling over?

Degree of Impact (0-100)

<PAGE BREAK>

8. Has **feeling dizzy** been affecting your ability to get a job or do your job?

O_1 Yes O_2 No

[SHOW IF YES] Please indicate on the scale; how much has feeling dizzy affected your ability to get a job or do your job?

Degree of Impact (0-100)

<PAGE BREAK>

9. Has **feeling dizzy** been making you afraid to going out?

O$_1$ Yes O$_2$ No

[SHOW IF YES] Please indicate on the scale; how much has feeling dizzy affected how afraid you are to go out?

O$_1$ Very little O$_2$ A little O$_3$ Moderately O$_4$ Very Much O$_5$ Severely

<PAGE BREAK>

10. Has **feeling dizzy** been affecting any other aspects of your daily functioning?

O$_1$ Yes O$_2$ No

[SHOW IF YES] Please describe the other aspect(s):

____

[SHOW IF YES] Please indicate on the scale; how much has feeling dizzy affected other aspects of your daily functioning?

Degree of Impact (0-100)

<PAGE BREAK>

11. **Feeling dizzy** has an impact on my functioning. ☐$_1$ True ☐$_2$ False

<PAGE BREAK>

12. How has **feeling dizzy** made you feel? (Please check all that apply – if any)

- ☐ Apathetic/Indifferent
- ☐ Frustrated
- ☐ Hopeless
- ☐ Dissatisfied
- ☐ Ashamed/Embarrassed
- ☐ Trusting/Accepting
- ☐ Overwhelmed
- ☐ Confused/Doubtful
- ☐ Impatient/Irritated/Angry
- ☐ Resigned
- ☐ None of the above **[EXCLUSIVE]**

<PAGE BREAK>

F. DIFFICULTY SLEEPING

[SKIP IF "NEVER" SELECTED FOR ITEM 23 OF GASS]

1. You have said that since taking your current medication that you **experienced difficulty sleeping**. How long has it been since you **experienced difficulty sleeping**?

O1 In the past day

O2 In the past week

O3 In the past month

O4 In the past 3 months

O5 In the past year

<PAGE BREAK>

2. Has **difficulty sleeping been** affecting your energy level?

O1 Yes O2 No

[SHOW IF YES] Please indicate on the scale; how much has difficulty sleeping affected your <u>energy level</u>? *(to enter your response below, click on the circle and drag your mouse to the left or right)*

Degree of Impact (0-100)

<PAGE BREAK>

3. Has **difficulty sleeping been** affecting your <u>level of physical comfort</u>?

O$_1$ Yes O$_2$ No

[SHOW IF YES] Please indicate on the scale; how much has difficulty sleeping affected your <u>level of physical discomfort</u>? *(to enter your response below, click on the circle and drag your mouse to the left or right)*

Degree of Impact (0-100)

<PAGE BREAK>

4. Has **difficulty sleeping been** affecting your <u>ability to do chores around the house</u>?

O$_1$ Yes O$_2$ No

[SHOW IF YES] Please indicate on the scale; how much has difficulty sleeping affected your <u>ability to do chores around the house</u>?

Degree of Impact (0-100)

<PAGE BREAK>

5. Has **difficulty sleeping been** affecting your ability to take care of yourself?

O_1 Yes O_2 No

[SHOW IF YES] Please indicate on the scale; how much has difficulty sleeping affected your ability to take care of yourself?

Degree of Impact (0-100)

<PAGE BREAK>

6. Has **difficulty sleeping been** affecting your avoiding or not talking to others?

O_1 Yes O_2 No

[SHOW IF YES] Please indicate on the scale; how much has difficulty sleeping affected how much you avoid others?

Degree of Impact (0-100)

<PAGE BREAK>

7. Has **difficulty sleeping been** making you afraid of going out?

O_1 Yes O_2 No

[SHOW IF YES] Please indicate on the scale; how much has difficulty sleeping affected how afraid you are to go out?

Degree of Impact (0-100)

<PAGE BREAK>

8. Has **difficulty sleeping been** affecting your ability to get a job or do your job?

O_1 Yes O_2 No

[SHOW IF YES] Please indicate on the scale; how much has difficulty sleeping affected your ability to get a job or do your job?

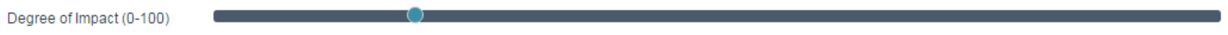

<PAGE BREAK>

9. Has **difficulty sleeping been** affecting your ability to concentrate?

O_1 Yes O_2 No

[SHOW IF YES] Please indicate on the scale; how much has difficulty sleeping affected your ability to concentrate?

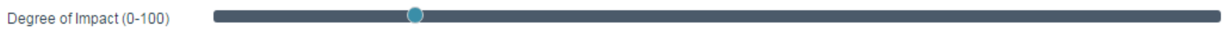

<PAGE BREAK>

10. Has **difficulty sleeping been** affecting your ability to follow classes?

O_1 Yes O_2 No

[SHOW IF YES] Please indicate on the scale; how much has difficulty sleeping affected your ability to follow classes?

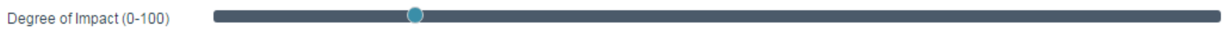

<PAGE BREAK>

11. Has **difficulty sleeping been** affecting your ability to drive?

O_1 Yes O_2 No

[SHOW IF YES] Please indicate on the scale; how much has difficulty sleeping affected your ability to drive?

Degree of Impact (0-100)

<PAGE BREAK>

12. Has **difficulty sleeping** been affecting <u>any other aspects of your daily functioning</u>?

O$_1$ Yes O$_2$ No

[SHOW IF YES] Please describe the other aspect(s):

____

[SHOW IF YES] Please indicate on the scale; how much has difficulty sleeping affected <u>other aspects of your daily functioning</u>?

Degree of Impact (0-100)

<PAGE BREAK>

13. **Difficulty sleeping** has an impact on my functioning. ☐$_1$ True ☐$_2$ False

<PAGE BREAK>

14. How has your **difficulty sleeping** made you feel? (Please check <u>all</u> that apply – if any)

- ☐ Apathetic/Indifferent
- ☐ Frustrated
- ☐ Hopeless
- ☐ Dissatisfied
- ☐ Ashamed/Embarrassed
- ☐ Trusting/Accepting
- ☐ Overwhelmed
- ☐ Confused/Doubtful
- ☐ Impatient/Irritated/Angry
- ☐ Resigned

☐ None of the above **[EXCLUSIVE]**

<PAGE BREAK>

G. PROBLEMS ENJOYING SEX

[<u>IF FEMALE</u>: SKIP IF "NEVER" SELECTED FOR ITEM 19 OF GASS; <u>IF MALE</u>: SKIP IF "NEVER" SELECTED FOR <u>BOTH</u> ITEMS 19 & 20 OF GASS]

1. You have said that since taking your current medication you have had problems enjoying sex. How long has it been since you experienced **problems enjoying sex?**

O_1 In the past day

O_2 In the past week

O_3 In the past month

O_4 In the past 3 months

O_5 In the past year

<PAGE BREAK>

2. *Have* **problems enjoying sex** been affecting your <u>physical discomfort</u>?

O_1 Yes O_2 No

[SHOW IF YES] Please indicate on the scale; how much has problems enjoying sex affected your <u>physical discomfort</u>? *(to enter your response below, click on the circle and drag your mouse to the left or right)*

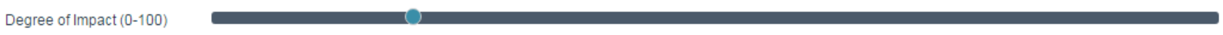

<PAGE BREAK>

3. *Have* **problems enjoying sex** been affecting <u>your ability to communicate with your partner</u>?

O_1 Yes O_2 No

[SHOW IF YES] Please indicate on the scale; how much has problems enjoying sex affected how much <u>your ability to communicate with your partner</u>? *(to enter your response below, click on the circle and drag your mouse to the left or right)*

Degree of Impact (0-100)

<PAGE BREAK>

4. *Have* **problems enjoying sex** been affecting your intimate relationships?

O$_1$ Yes O$_2$ No

[SHOW IF YES] Please indicate on the scale; how much has problems enjoying sex affected <u>your intimate relationships</u>?

Degree of Impact (0-100)

<PAGE BREAK>

5. Have **problems enjoying sex** been affecting <u>any other aspects of your daily functioning</u>?

O$_1$ Yes O$_2$ No

[SHOW IF YES] Please describe the other aspect(s):

____

[SHOW IF YES] Please indicate on the scale; how much have problems having sex affected <u>other aspects of your daily functioning</u>?

Degree of Impact (0-100)

<PAGE BREAK>

6. **Problems enjoying sex** has an impact on my functioning. ☐$_1$ True ☐$_2$ False

<PAGE BREAK>

7. How have your **problems enjoying sex** made you feel? (Please check <u>all</u> that apply – if any)

- ☐ Apathetic/Indifferent
- ☐ Frustrated
- ☐ Hopeless
- ☐ Dissatisfied
- ☐ Ashamed/Embarrassed
- ☐ Trusting/Accepting
- ☐ Overwhelmed
- ☐ Confused/Doubtful
- ☐ Impatient/Irritated/Angry
- ☐ Resigned
- ☐ Self-conscious
- ☐ Worthless
- ☐ Feel a lack of love
- ☐ None of the above **[EXCLUSIVE]**

<PAGE BREAK>

H. WEIGHT GAIN

[SKIP IF "NEVER" SELECTED FOR ITEM 22 OF GASS]

1. You have said that since taking your current medication you have had problems with weight gain. How long has it been since you experienced weight gain?

O_1 In the past day

O_2 In the past week

O_3 In the past month

O_4 In the past 3 months

O_5 In the past year

<PAGE BREAK>

2. Has **your weight gain** been affecting your energy level?

O_1 Yes O_2 No

[SHOW IF YES] Please indicate on the scale; how much has weight gain affected your energy level? *(to enter your response below, click on the circle and drag your mouse to the left or right)*

Degree of Impact (0-100)

<PAGE BREAK>

3. Has **your weight gain** affected your ability to put on or take off clothing?

O_1 Yes O_2 No

[SHOW IF YES] Please indicate on the scale; how much has **weight gain** affected your ability to put on or take off clothing? *(to enter your response below, click on the circle and drag your mouse to the left or right)*

Degree of Impact (0-100)

<PAGE BREAK>

4. Has **your weight gain** affected your ability to ability to do chores around the house?

O_1 Yes O_2 No

[SHOW IF YES] Please indicate on the scale; how much has **weight gain** affected your ability to do chores around the house?

Degree of Impact (0-100)

<PAGE BREAK>

5. Has **your weight gain** affected your ability to ability to take care of yourself?

O_1 Yes O_2 No

[SHOW IF YES] Please indicate on the scale; how much has **weight gain** affected your ability to take care of yourself?

Degree of Impact (0-100)

<PAGE BREAK>

6. Has **your weight gain** affected your fear of being rejected?

O_1 Yes O_2 No

[SHOW IF YES] Please indicate on the scale; how much has weight gain affected your fear of being rejected?

Degree of Impact (0-100)

<PAGE BREAK>

7. Has **your weight gain** affected how afraid you are to go out?

O_1 Yes O_2 No

[SHOW IF YES] Please indicate on the scale; how much has **weight gain** affected how afraid you are to go out?

Degree of Impact (0-100)

<PAGE BREAK>

8. Has **your weight gain** caused you to experience ridicule, teasing, or unwanted attention?

O_1 Yes O_2 No

[SHOW IF YES] Please indicate on the scale; how much has weight gain caused you to experience ridicule, teasing, or unwanted attention?

Degree of Impact (0-100)

<PAGE BREAK>

9. Has **your weight gain** affected your ability to meet responsibilities or given you difficulty in getting things done?

O_1 Yes O_2 No

[SHOW IF YES] Please indicate on the scale; how much has weight gain affected your ability to meet responsibilities or given you difficulty in getting things done?

Degree of Impact (0-100)

<PAGE BREAK>

10. Has **your weight gain** affected your ability to have an intimate relationship?

O_1 Yes O_2 No

[SHOW IF YES] Please indicate on the scale; how much has weight gain affected your ability to have an intimate relationship?

Degree of Impact (0-100)

<PAGE BREAK>

11. Has **your weight gain** been affecting any other aspects of your daily functioning?

O_1 Yes O_2 No

[SHOW IF YES] Please describe the other aspect(s):

____

[SHOW IF YES] Please indicate on the scale; how much has weight gain affected <u>other aspects of your daily functioning</u>?

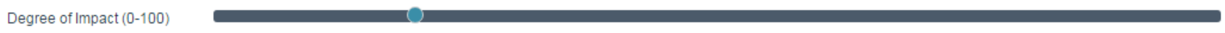

<PAGE BREAK>

12. **Weight gain** has an impact on my functioning. ☐$_1$ True ☐$_2$ False

<PAGE BREAK>

13. How has your weight gain made you feel? (Please check <u>all</u> that apply – if any)

- ☐ Lack of confidence
- ☐ Feel Worthless
- ☐ Apathetic/Indifferent
- ☐ Frustrated
- ☐ Hopeless
- ☐ Dissatisfied
- ☐ Ashamed/Embarrassed
- ☐ Trusting/Accepting
- ☐ Overwhelmed
- ☐ Confused/Doubtful
- ☐ Impatient/Irritated/Angry
- ☐ Resigned
- ☐ I was trying to gain weight, so it was not an issue for me
- ☐ None of the above **[EXCLUSIVE]**
