# Supplementary material for: The impact on functioning of second-generation antipsychotic medication side effects for patients with schizophrenia: a worldwide, cross-sectional, web-based survey
Source: Ann Gen Psychiatry. 2020 Jul 13;19:42. doi: 10.1186/s12991-020-00292-5 (PMC7359579; doi:10.1186/s12991-020-00292-5)
Supplement: Supplementary file 2 — Additional file 2. Impact of Side Effects on Functioning. [file 12991_2020_292_MOESM2_ESM.pdf]

Impact of Side Effects on Functioning

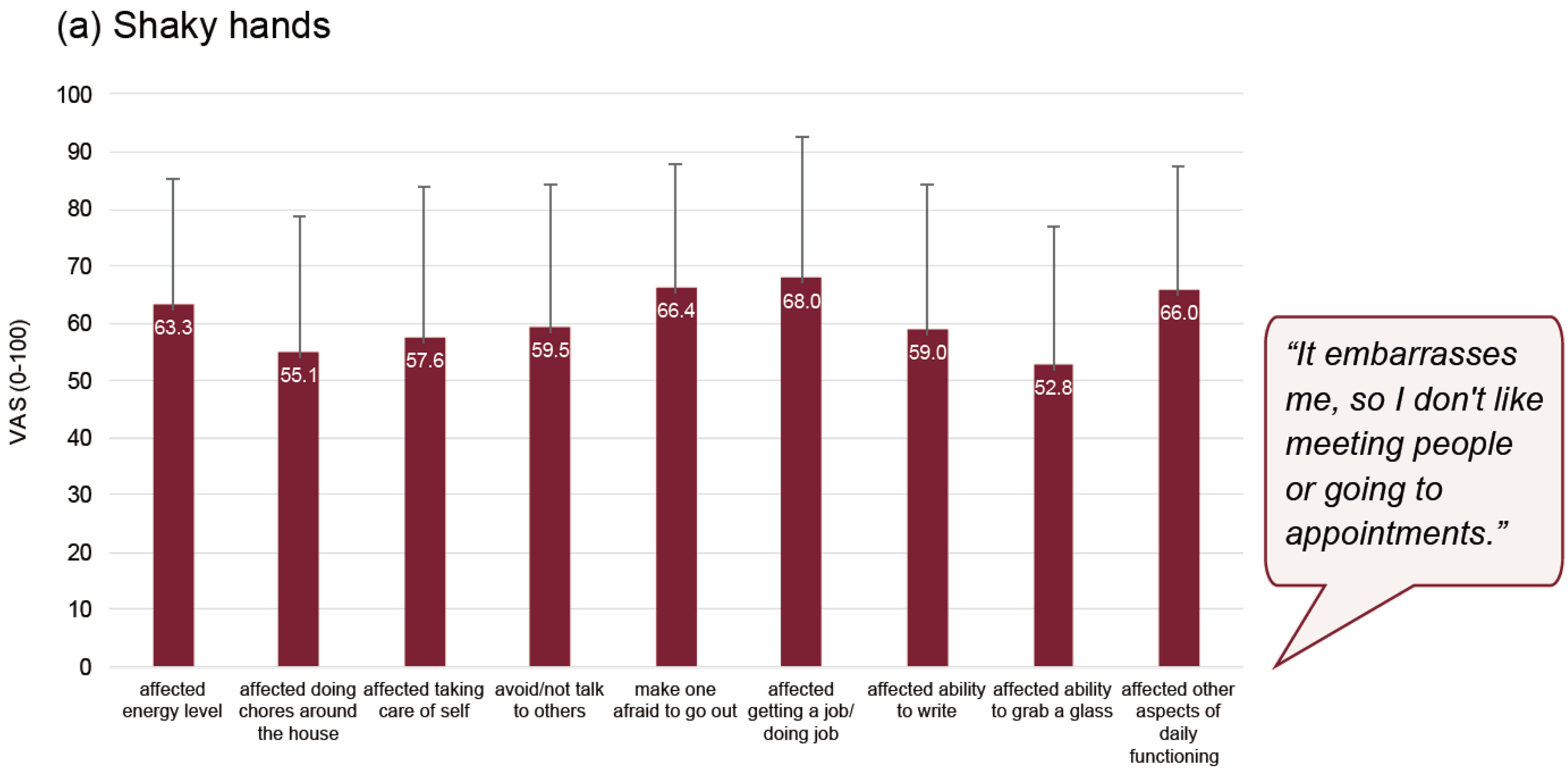

Impact of Side Effects on Functioning

(b) Restlessness

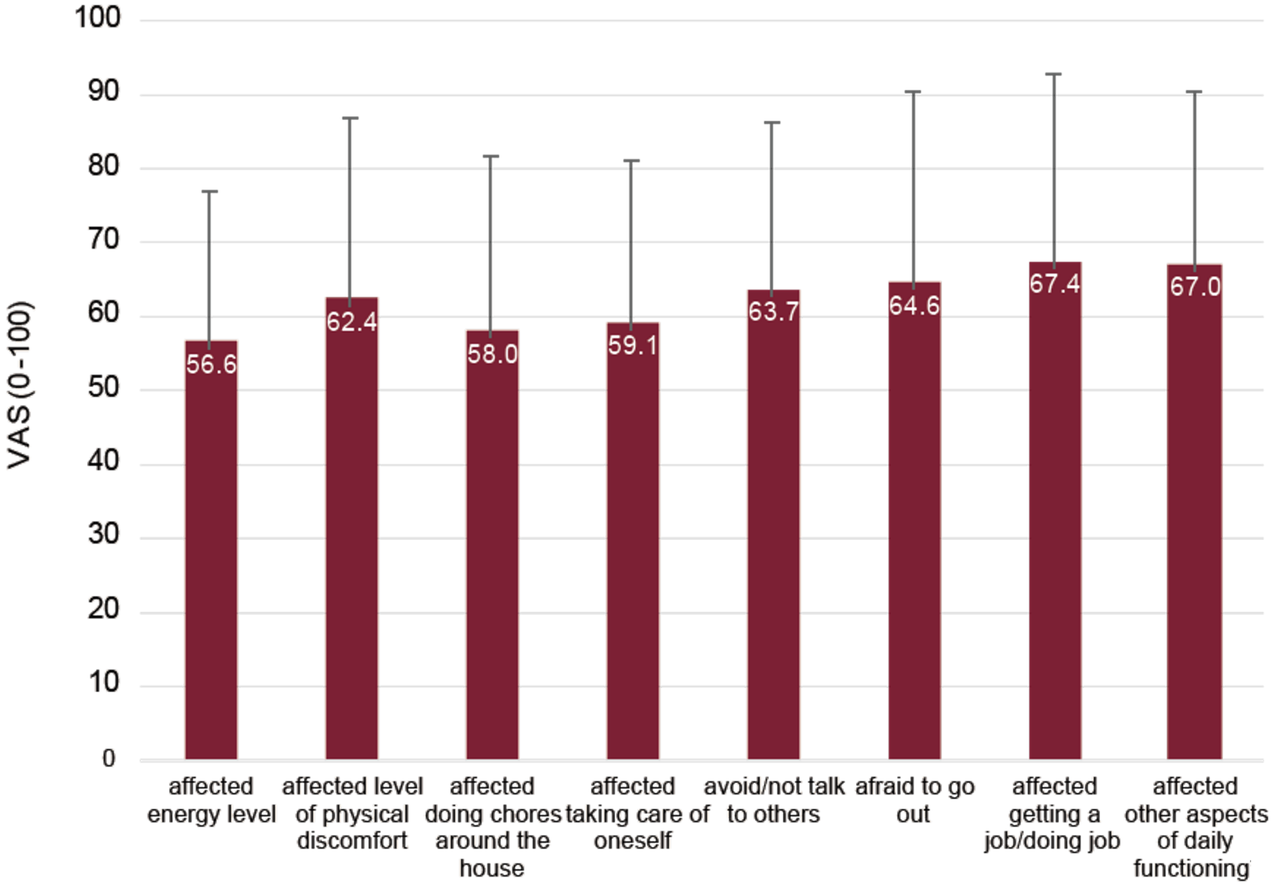

> *"It creates (gives) me anxiety and I can not under-stand why, it scares me and prevents me from continuing to do any work."*

Impact of Side Effects on Functioning

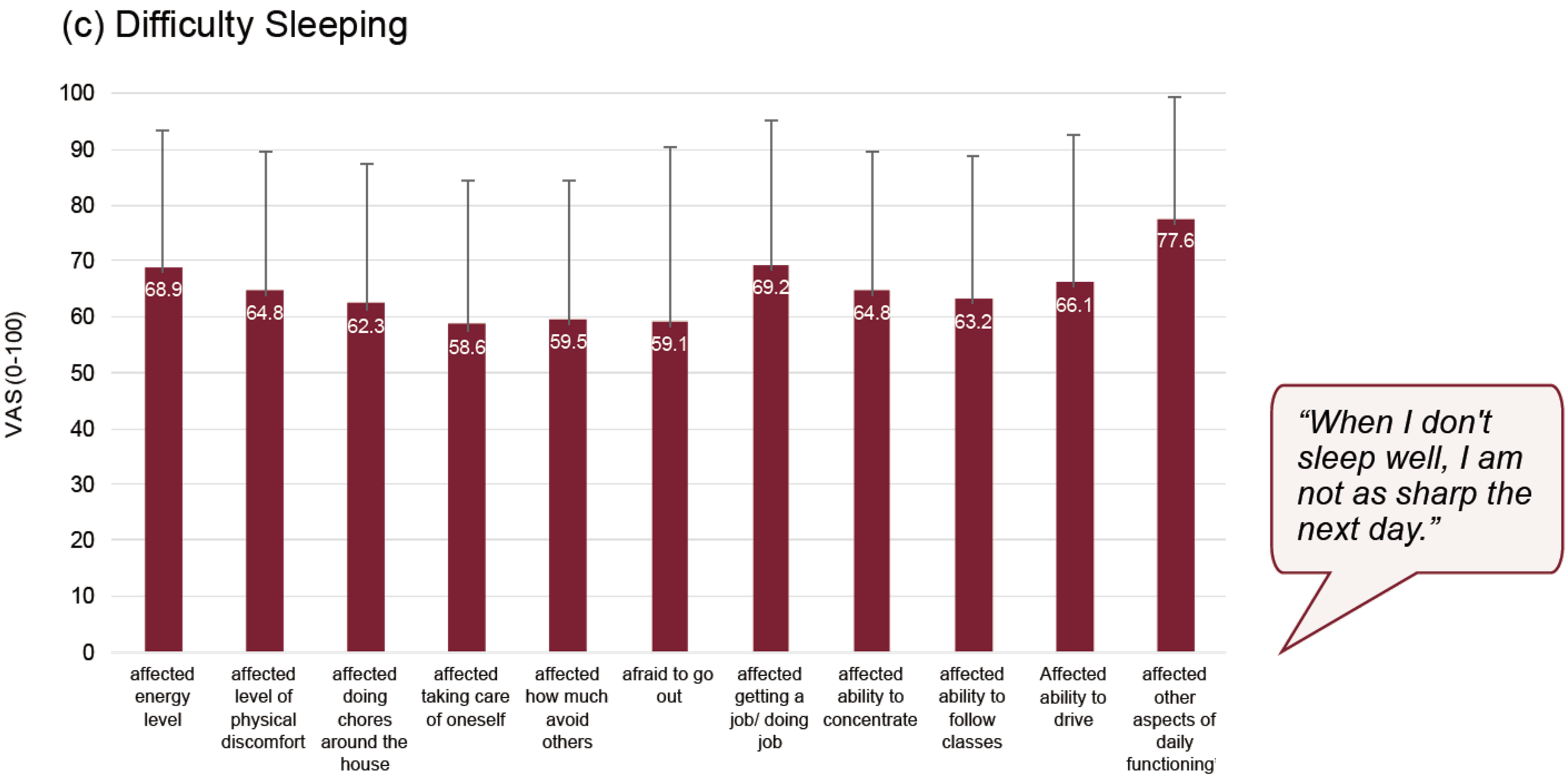

Impact of Side Effects on Functioning

(d) Sleepiness during the day

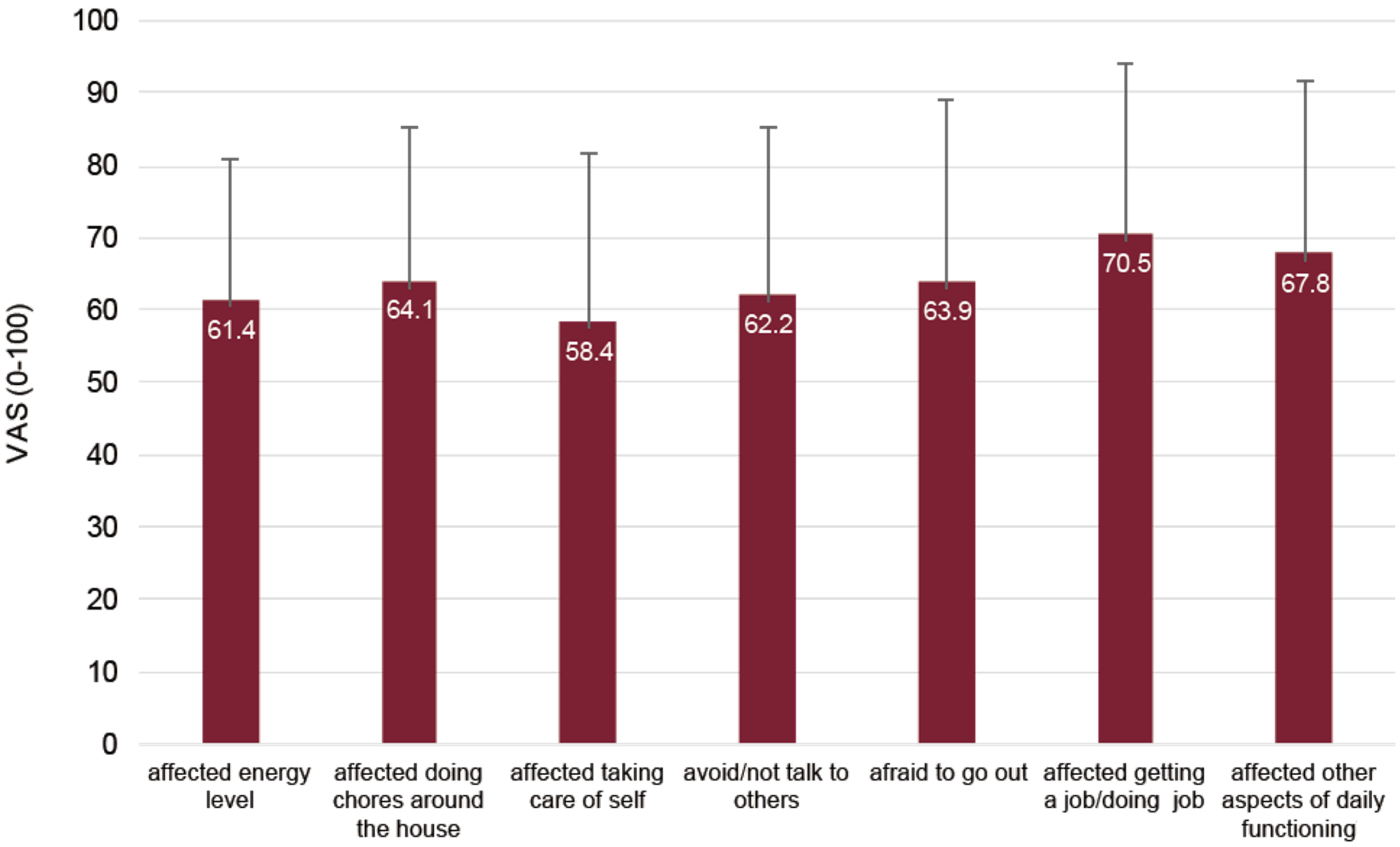

"I just want to sleep and I don't want to do any-thing."

Impact of Side Effects on Functioning

(e) Feeling Drugged or Like a Zombie

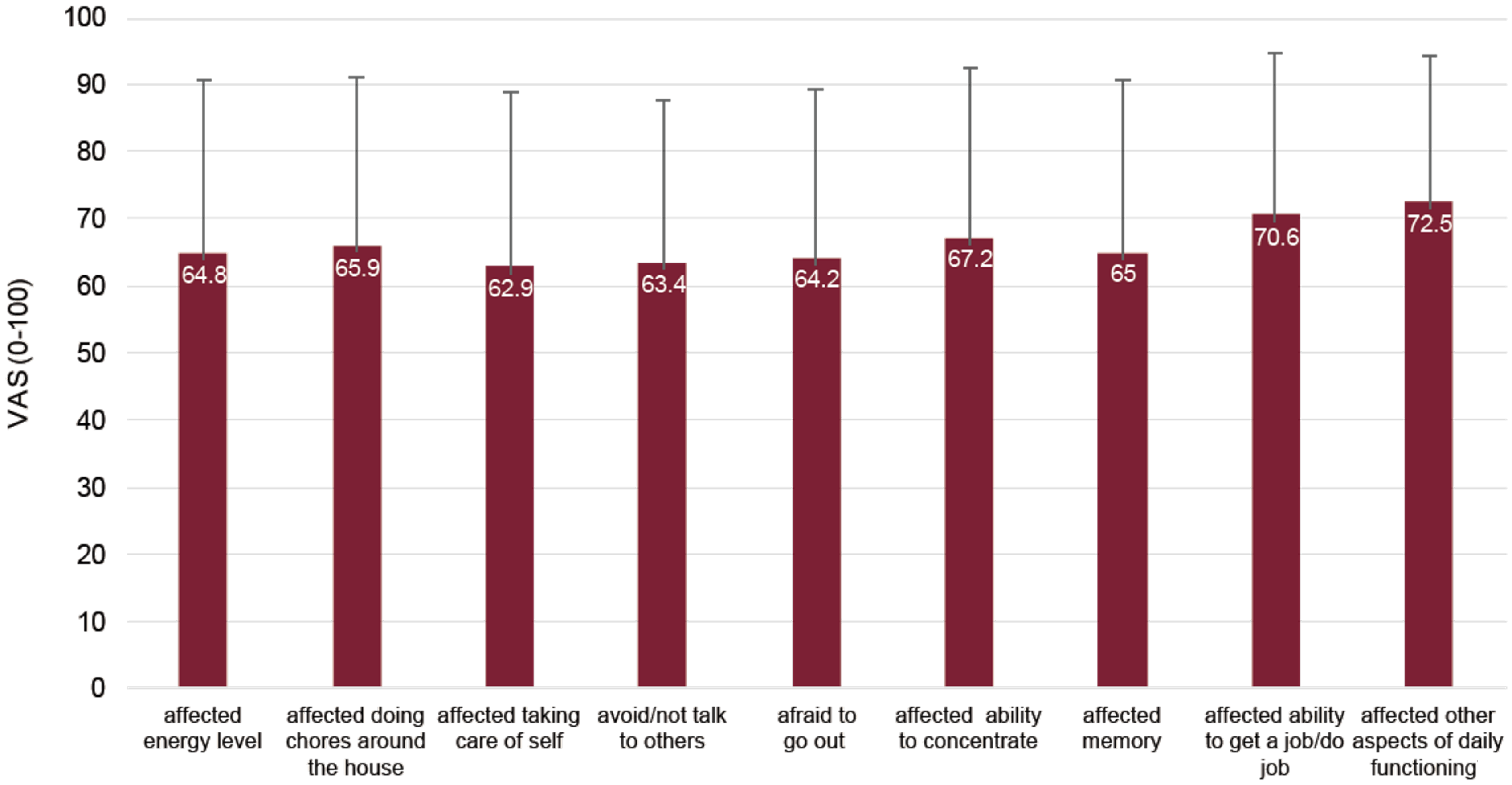

"It's difficult to perform everyday things like washing, cooking, etc."

Impact of Side Effects on Functioning

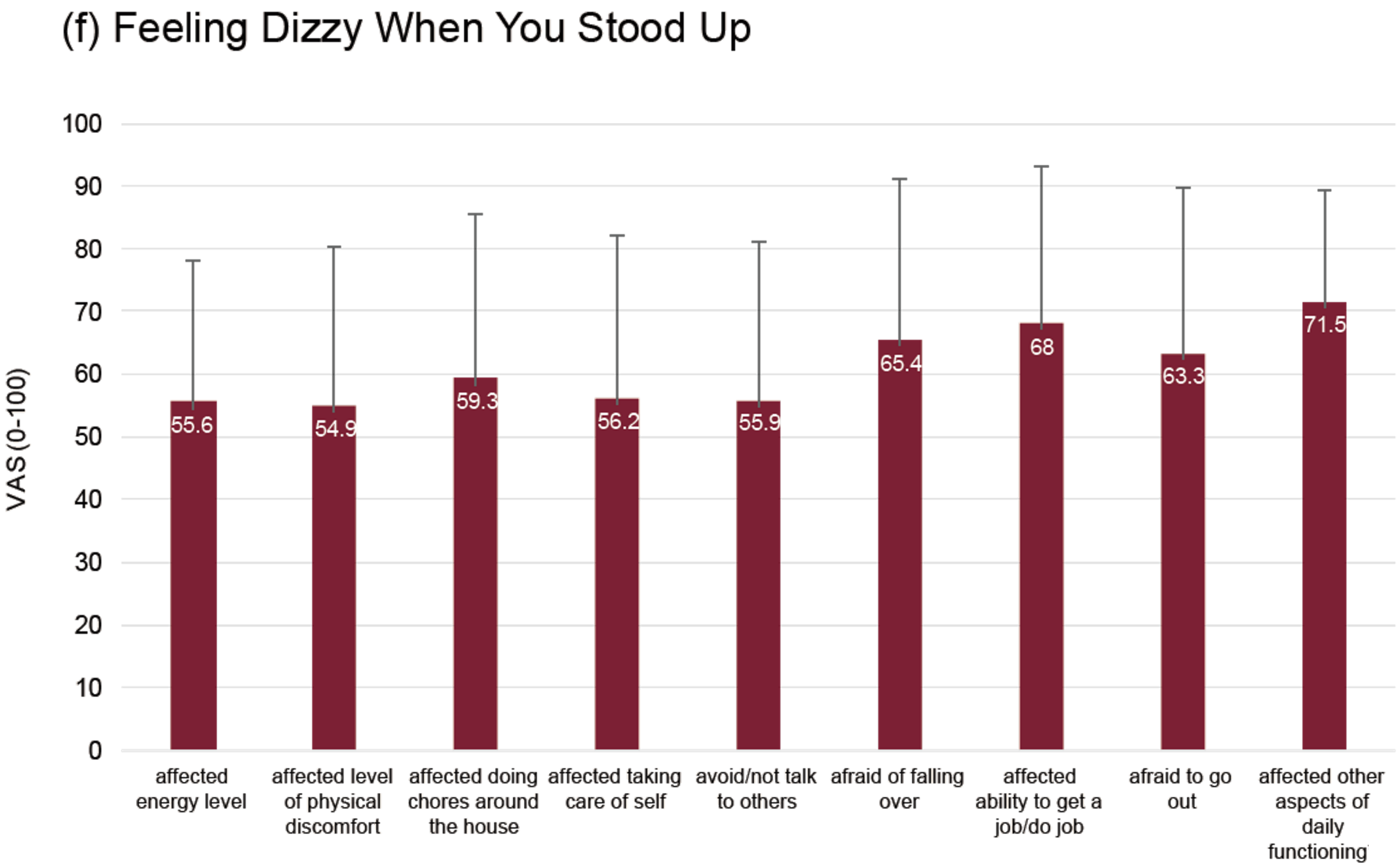

"I have difficulties to plan the jobs to do because my head is spinning and I don't feel safe."

Impact of Side Effects on Functioning

(g) Problems Enjoying Sex

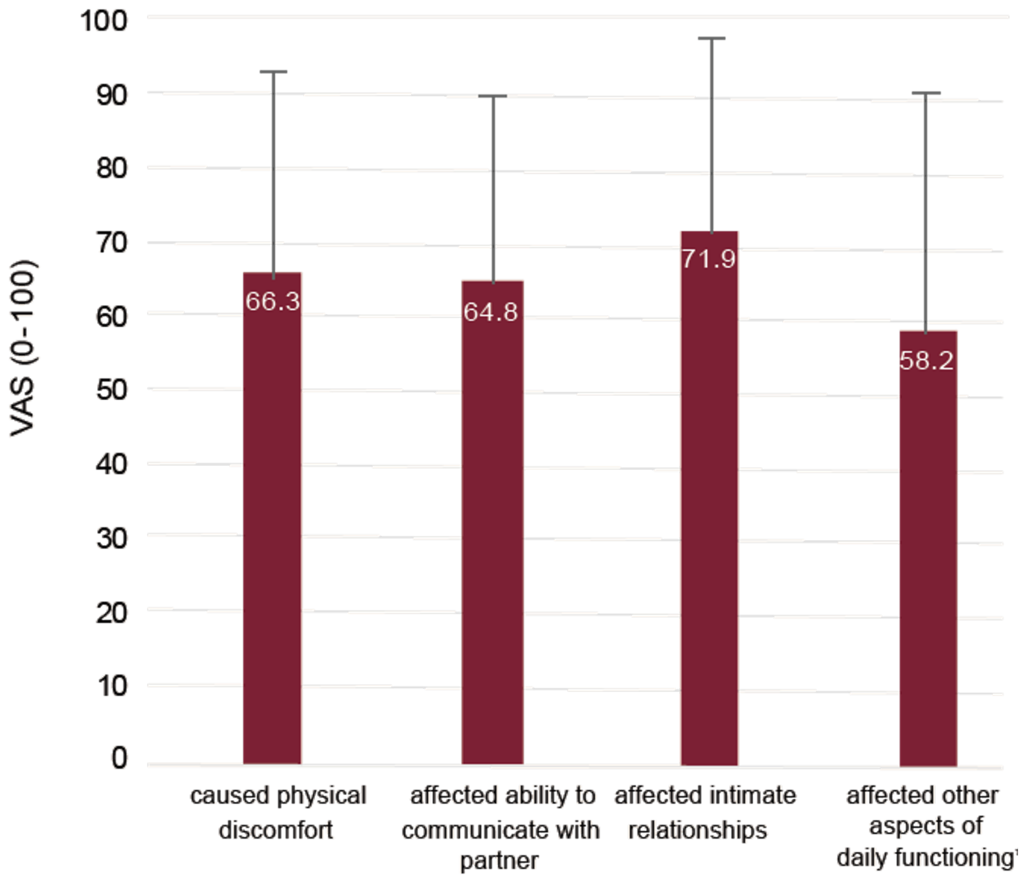

"I no longer feel to be a man, I do not trust myself, I no longer have my virility and I feel frustrated, use-less, done for as if my life had stopped, the ugliest thing is my resignation to this situation."

Impact of Side Effects on Functioning

(h) Gaining weight

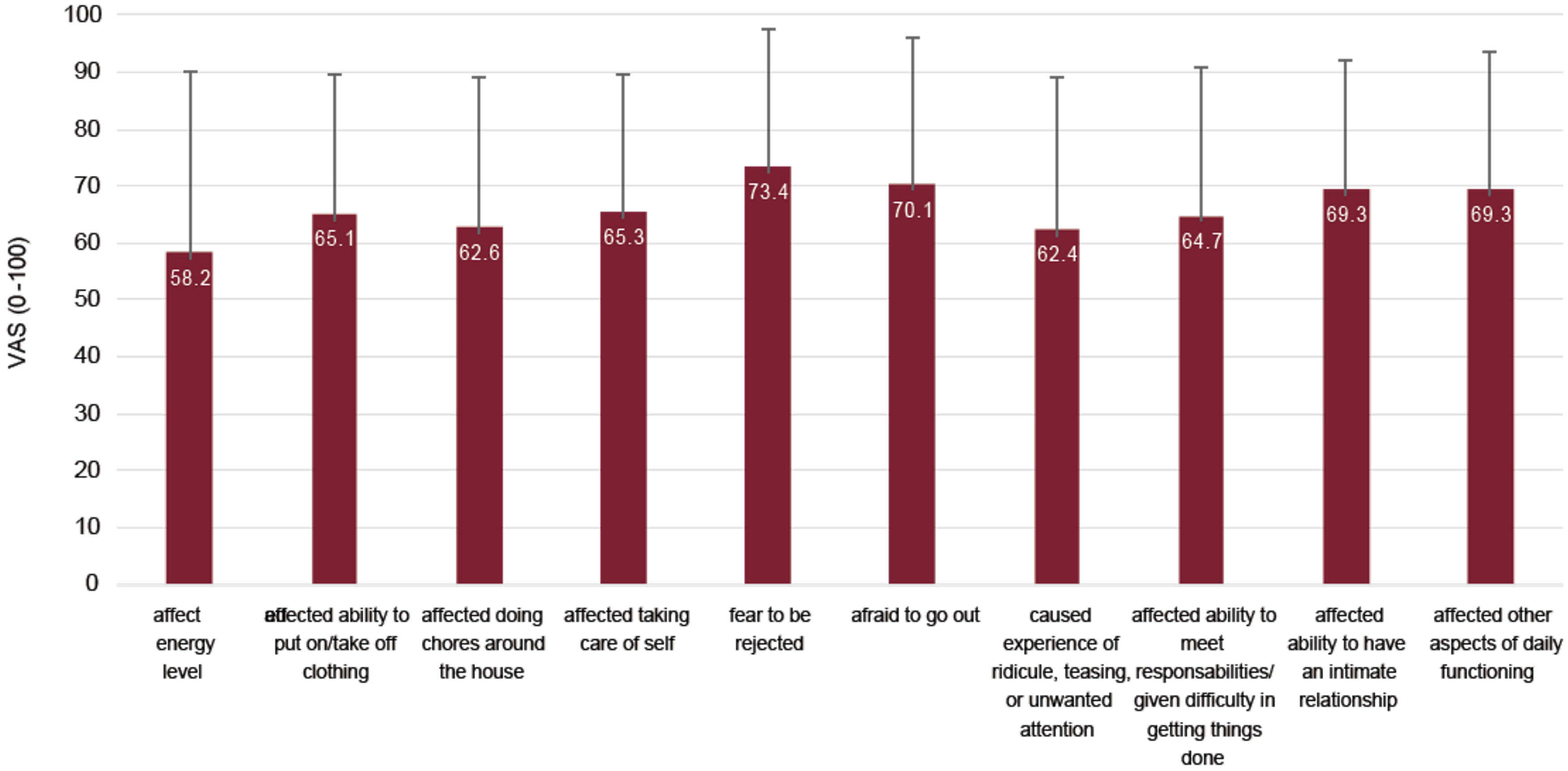

"It is hard for me to walk, go up stairs, in my wardrobe I have clothes of all sizes because I still hope to lose weight some day."
