# Supplementary material for: The impact on functioning of second-generation antipsychotic medication side effects for patients with schizophrenia: a worldwide, cross-sectional, web-based survey
Source: Ann Gen Psychiatry. 2020 Jul 13;19:42. doi: 10.1186/s12991-020-00292-5 (PMC7359579; doi:10.1186/s12991-020-00292-5)
Supplement: Supplementary file 3 — Additional file 3. Table a. Sample characteristics by region. Table b. Mean Side Effect Scores as measured by the Glasgow Antipsychotic Side-Effect Scale (GASS), All Countries Combined by Age and Overall. Table c. Mean Side Effect Scores as measured by the Glasgow Antipsychotic Side-Effect Scale (GASS), All Countries Combined by Gender and Overall. Table d. Mean Severity (VAS Scales) of Key Side Effects’ Impact on Functioning (Subset analysis), All Countries Combined by Employment Status and Overall. Table e. Linear Regression Model of Activating Side Effects, Demographics, and Time Since Diagnosis on HRQoL (Model A). Table f. Linear Regression Model of Sedating Side Effects, Demographics, and Time Since Diagnosis on HRQoL (Model B). Table g. Linear Regression Linear Regression Model of Other Side Effects, Demographics, and Time Since Diagnosis on HRQoL (Model C). Table h. Quality of Life Enjoyment and Satisfaction Questionnaire Short Form (Q-LES-Q-SF) Item Descriptive Statistics, All Countries Combined by Gender and Overall. Table i. Quality of Life Enjoyment and Satisfaction Questionnaire Short Form (Q-LES-Q-SF) Item Descriptive Statistics, All Countries Combined by Employment Status and Overall [file 12991_2020_292_MOESM3_ESM.pdf]

ADDITIONAL FILE 3

Table a. Sample characteristics by region

	US (N=180)	Canada (N=99)	Australia (N=28)	EU Countries (N=128)
**Male sex, n (%)**	**75 (41.7%)**	**58 (58.6%)**	**11 (39.3%)**	**54 (42.2%)**
**Age (years); mean (SD)**	35.3 (9.0)	33.1 (9.1)	35.9 (11.3)	45.8 (10.8)
**Ethnicity, n (%)**				
White	69.4%	56.6%	60.7%	Not asked
Other	30.6%	43.4%	39.3%	
**Living Situation, n (%)**				
Parent(s)	32 (17.8%)	19 (19.2%)	8 (28.6%)	30 (23.4%)
Spouse/partner without children	52 (28.9%)	11 (11.1%)	1 (3.6%)	33 (25.8%)
Spouse/partner with children	53 (29.4%)	23 (23.2%)	8 (28.6%)	31 (24.2%)
Children without spouse/partner	10 (5.6%)	4 (4.0%)	1 (3.6%)	4 (3.1%)
Other family members	3 (1.7%)	3 (3.0%)	1 (3.6%)	7 (5.5%)
Group home or community facility staff	4 (2.2%)	0 (0.0%)	1 (3.6%)	11 (8.6%)
Alone	20 (11.1%)	33 (33.3%)	7 (25.0%)	10 (7.8%)
Other	5 (2.8%)	6 (6.1%)	1 (3.6%)	2 (1.6%)
**Employment status, n (%)**				
Employed, full time or part time	58 (32.2%)	54 (54.5%)	13 (46.4%)	48 (37.5%)
Student	10 (5.6%)	12 (12.1%)	1 (3.6%)	1 (0.8%)
Volunteer	1 (0.6%)	4 (4.0%)	3 (10.7%)	4 (3.1%)
Unemployed	33 (18.3%)	1 (1.0%)	3 (10.7%)	23 (18.0%)
Retired	4 (2.2%)	7 (7.1%)	0 (0.0%)	3 (2.3%)
Disabled and able to work	15 (8.3%)	4 (4.0%)	1 (3.6%)	13 (10.2%)
Disabled and unable to work	31 (17.2%)	12 (12.1%)	2 (7.1%)	9 (7.0%)
Homemaker	28 (15.6%)	5 (5.1%)	5 (17.9%)	27 (21.1%)
**Education level, n (%)**				
Elementary/primary school	5 (2.8%)	0 (0.0%)	1 (3.6%)	15 (11.7%)
Secondary/high school	44 (24.4%)	18 (18.2%)	6 (21.4%)	55 (43.0%)
Some college	67 (37.2%)	23 (23.2%)	3 (10.7%)	7 (5.5%)
College degree	23 (12.8%)	32 (32.3%)	12 (42.9%)	11 (8.6%)
Some graduate school	16 (8.9%)	7 (7.1%)	1 (3.6%)	14 (10.9%)
Graduate degree	11 (6.1%)	14 (14.1%)	1 (3.6%)	12 (9.4%)
Technical or vocational school	10 (5.6%)	2 (2.0%)	4 (14.3%)	7 (5.5%)
Other	4 (2.2%)	3 (3.0%)	0 (0.0%)	7 (5.5%)

Table b. Mean Side Effect Scores as measured by the Glasgow Antipsychotic Side-Effect Scale (GASS), All Countries Combined by Age and Overall

Q-LES-Q-SF Items	Younger: 37 or less (N=232)		Older: 38 years or more (N=202)		p-value[1]	Overall (N=434)	
	N	Mean (SD)	N	Mean (SD)		N	Mean (SD)
Felt sleepy during the day	232	1.9 (1.0)	201	1.7 (1.0)	0.0418	433	1.8 (1.0)
Felt drugged like a zombie	232	1.2 (1.1)	202	0.8 (1.0)	0.0002	434	1.0 (1.1)
Felt dizzy when standing up	231	1.1 (1.0)	201	0.8 (1.0)	0.0015	432	0.9 (1.0)
Felt heart beating irregularly	231	1.0 (1.0)	201	1.1 (1.0)	0.4926	432	1.0 (1.0)
Muscles tense or jerky	229	1.1 (1.1)	202	1.0 (1.1)	0.3254	431	1.0 (1.1)
Hands or arms been shaky	230	1.0 (1.0)	200	0.8 (1.0)	0.1253	430	0.9 (1.0)
Legs felt restless/couldn't sit still	230	1.2 (1.1)	201	0.8 (1.0)	<.0001	431	1.0 (1.1)
Have been drooling	229	0.5 (0.9)	201	0.5 (0.8)	0.5538	430	0.5 (0.9)
Movements or walking been slower	229	0.9 (1.0)	202	0.9 (1.1)	0.9083	431	0.9 (1.0)
Uncontrollable movements of face or body	231	0.6 (0.9)	202	0.5 (0.9)	0.6225	433	0.5 (0.9)
Vision has been blurry	232	1.1 (1.0)	202	0.7 (1.0)	<.0001	434	0.9 (1.0)
Mouth has been dry	231	1.3 (1.1)	201	1.3 (1.1)	0.7446	432	1.3 (1.1)
Difficulty passing urine	231	0.5 (0.8)	202	0.5 (0.9)	0.7680	433	0.5 (0.9)
Going to be sick or vomit	231	0.8 (1.0)	201	0.6 (0.8)	0.0169	432	0.7 (0.9)
Wet the bed	232	0.3 (0.7)	201	0.3 (0.7)	0.3654	433	0.3 (0.7)
Very thirsty/passing urine	231	1.1 (1.1)	201	1.0 (1.1)	0.4296	432	1.1 (1.1)
Sore or swollen nipples	231	0.4 (0.8)	200	0.4 (0.8)	0.7531	431	0.4 (0.8)
Fluid coming from nipples	232	0.2 (0.6)	201	0.3 (0.7)	0.4638	433	0.3 (0.7)
Problems enjoying sex	231	1.1 (1.1)	200	1.0 (1.1)	0.8471	431	1.1 (1.1)
Problems getting erection[2]	109	0.6 (1.0)	91	0.8 (1.0)	0.1427	200	0.7 (1.0)
Change in periods (Yes-1/No-2)[3]	123	1.6 (0.5)	110	1.7 (0.4)	0.0679	233	1.7 (0.5)
Gaining weight (Yes-1/No-2)	226	1.5 (0.5)	200	1.5 (0.5)	0.7040	426	1.5 (0.5)
Experienced difficulty sleeping	232	1.6 (1.0)	202	1.4 (1.1)	0.0438	434	1.5 (1.0)

Abbreviations: SD = standard deviation. Higher scores indicate more severe side effects. [1] P-values- One-way ANOVA test for continuous variables. Chi-square used for categorical variables. [2] Only asked to males. [3] Only asked to females.

Table c. Mean Side Effect Scores as measured by the Glasgow Antipsychotic Side-Effect Scale (GASS), All Countries Combined by Gender and Overall

Q-LES-Q-SF Items	Females (N=234)		Males (N=198)		p-value[1]	Overall (N=432)	
	N	Mean (SD)	N	Mean (SD)		N	Mean (SD)
Felt sleepy during the day	233	2.0 (1.0)	198	1.6 (1.0)	<.0001	431	1.8 (1.0)
Felt drugged like a zombie	234	1.1 (1.1)	198	1.0 (1.1)	0.3363	432	1.0 (1.1)
Felt dizzy when standing up	232	1.0 (1.0)	198	0.9 (1.0)	0.2723	430	0.9 (1.0)
Felt heart beating irregularly	232	1.1 (1.1)	198	0.9 (1.0)	0.0354	430	1.0 (1.0)
Muscles tense or jerky	233	1.1 (1.1)	197	0.9 (1.0)	0.0906	430	1.0 (1.1)
Hands or arms been shaky	231	0.9 (1.0)	198	0.8 (1.0)	0.2401	429	0.9 (1.0)
Legs felt restless/couldn't sit still	232	1.1 (1.1)	198	0.9 (1.1)	0.2251	430	1.0 (1.1)
Have been drooling	232	0.5 (0.9)	197	0.5 (0.9)	0.8751	429	0.5 (0.9)
Movements or walking been slower	233	1.0 (1.1)	197	0.8 (1.0)	0.0121	430	0.9 (1.0)
Uncontrollable movements of face or body	233	0.6 (1.0)	198	0.5 (0.8)	0.1220	431	0.5 (0.9)
Vision has been blurry	234	1.0 (1.1)	198	0.8 (1.0)	0.0197	432	0.9 (1.0)
Mouth has been dry	232	1.3 (1.2)	198	1.2 (1.0)	0.2566	430	1.3 (1.1)
Difficulty passing urine	233	0.4 (0.8)	198	0.6 (0.9)	0.1248	431	0.5 (0.9)
Going to be sick or vomit	233	0.8 (1.0)	198	0.6 (0.9)	0.0269	431	0.7 (0.9)
Wet the bed	233	0.3 (0.7)	198	0.3 (0.7)	0.6756	431	0.3 (0.7)
Very thirsty/passing urine	233	1.2 (1.2)	198	0.9 (1.0)	0.0312	431	1.1 (1.1)
Sore or swollen nipples	232	0.5 (0.9)	198	0.3 (0.7)	0.1199	430	0.4 (0.8)
Fluid coming from nipples	233	0.3 (0.7)	198	0.2 (0.5)	0.0239	431	0.3 (0.6)
Problems enjoying sex	231	1.2 (1.2)	198	0.9 (1.0)	0.0014	429	1.1 (1.1)
Problems getting erection[2]			198	0.7 (1.0)		198	0.7 (1.0)
Change in periods (Yes-1/No-2)[3]	230	1.7 (0.5)				230	1.7 (0.5)
Gaining weight (Yes-1/No-2)	230	1.4 (0.5)	194	1.5 (0.5)	0.0019	424	1.5 (0.5)
Experienced difficulty sleeping	234	1.6 (1.1)	198	1.5 (1.0)	0.2953	432	1.5 (1.0)

Abbreviations: SD = standard deviation. Higher scores indicate more severe side effects.
[1] P-values- One-way ANOVA test for continuous variables. Chi-square used for categorical variables. [2] Only asked to males.[3] Only asked to females.

Table d. Mean Severity (VAS Scales) of Key Side Effects' Impact on Functioning (Subset analysis), All Countries Combined by Employment Status[a] and Overall

Impact	Side-Effect Category	Employed Mean Severity Scores, Mean (SD) (n)	Unemployed Mean Severity Scores, Mean (SD) (n)	Other Mean Severity Scores, Mean (SD) (n)	Overall Mean Severity Scores, Mean (SD) (n)
Shaky Hands or Arms	Activating	51.0 (21.8) (n=78)	60.4 (19.6) (n=43)	57.2 (21.5) (n=28)	54.8 (21.4) (n=149)
Restlessness	Activating	52.5 (20.3) (n=99)	64.8 (19.3) (n=52)	60.0 (22.1) (n=30)	57.3 (21.0) (n=181)
Difficulty Sleeping	Activating	55.3 (22.1) (n=151)	65.4 (21.4) (n=70)	62.2 (22.0) (n=54)	59.2 (22.3) (n=275)
Sleepy During the Day	Sedating	55.2 (18.3) (n=176)	65.7 (18.7) (n=83)	61.8 (18.5) (n=68)	59.2 (18.9) (n=327)
Feeling Dizzy/Fainted	Sedating	52.3 (23.2) (n=88)	57.9 (22.4) (n=49)	52.9 (22.6) (n=43)	54.0 (22.8) (n=180)
Feeling Drugged or like a Zombie	Sedating	57.0 (22.1) (n=117)	67.4 (24.3) (n=54)	62.2 (20.4) (n=43)	60.7 (22.7) (n=214)
Problems Enjoying Sex	Other	61.0 (24.8) (n=93)	70.8 (22.6) (n=60)	66.7 (23.9) (n=32)	65.2 (24.2) (n=185)
Gaining Weight	Other	57.3 (21.3) (n=101)	71.0 (19.1) (n=46)	62.0 (23.5) (n=34)	61.7 (21.9) (n=181)

[a] Employed= Full or part time work or disabled and able to work; Unemployed= Unemployed or Disabled and unable to work; Other=Student, Volunteer, Retired, or Homemaker. Percent completed of total sample, of those who reported the side-effect on GASS. Subset analysis based on whether side-effect of interest is reported on the GASS scale at least once. "Activating" side effects refer to restlessness, tremor, and difficulty sleeping; "sedating" refers to sleepiness, feeling drugged/like a zombie, and dizziness. "Other" refers to weight gain and problems enjoying sex. Participants were able to skip questions in the e-survey, thus missing data are expected. Defining table: The mean visual analogue scale (VAS) scores across each side effect (0-100).

Table e. Linear Regression Model of Activating Side Effects, Demographics, and Time Since Diagnosis on HRQoL (Model A): (N=435)

**Predictor: Single GASS Side Effect Category**	**Q-LES-Q-SF Total (N=435) Multivariate Estimate (SE)**
GASS Activating Side Effects	-3.15 (0.75)*
Age (continuous)	-0.13 (0.07)*
Gender (Female=1; Male=0)	-2.82 (1.20)*
Race/ Ethnicity (White=1; Other=0)	1.13 (1.85)
Education (At least Some College=1, No College=0)	0.27 (1.26)
Employment status (Employed Full or Part time=1, Not Employed=0)	1.32 (1.21)
Living situation (Live Alone=1; Live with Others=0)	-1.02 (1.49)
Time since diagnosis	0.45 (0.66)

*Abbreviations: SE, Standard Error; GASS = Glasgow Antipsychotic Side-Effect Scale; HRQoL, health-related quality of life; Q-LES-Q-SF = Quality of Life Enjoyment and Satisfaction Questionnaire-Short Form. Countries include Denmark, Norway, Spain, Italy, USA, Canada, and Australia. "Activating" Side Effects refer to "Restlessness," "Tremor," and "Difficulty sleeping". * Denotes p-value significant at p <0.005.*

Table f. Linear Regression Model of Sedating Side Effects, Demographics, and Time Since Diagnosis on HRQoL (Model B): (N=435)

**Predictor: Single GASS Side Effect Category**	**Q-LES-Q-SF Total (N=435) Multivariate Estimate (SE)**
GASS Sedating Side Effects	-4.67 (0.78)*
Age (continuous)	-0.17 (0.07)
Gender (Female=1; Male=0)	-2.56 (1.16)*
Race/ Ethnicity (White=1; Other=0)	0.72 (1.80)
Education (At least Some College=1, No College=0)	0.43 (1.21)
Employment status (Employed Full or Part time=1, Not Employed=0)	1.59 (1.16)
Living situation (Live Alone=1; Live with Others=0)	-0.49 (1.45)
Time since diagnosis	-0.05 (0.64)

*Abbreviations: GASS = Glasgow Antipsychotic Side-Effect Scale; HRQoL, health-related quality of life; Q-LES-Q-SF = Quality of Life Enjoyment and Satisfaction Questionnaire-Short Form. Countries include Denmark, Norway, Spain, Italy, USA, Canada, and Australia. "Sedating" refers to "Sleepiness during the day," Dizziness," and "Feeling drugged/like a zombie". * Denotes p-value significant at p <0.005.*

Table g Linear Regression Linear Regression Model of Other Side Effects, Demographics, and Time Since Diagnosis on HRQoL (Model C): (N=435)

Predictor: Single GASS Side Effect Category	Q-LES-Q-SF Total (N=435) Multivariate Estimate (SE)
GASS Other Side Effects	-3.06 (0.73)*
Age (continuous)	-0.12 (0.07)
Gender (Female=1; Male=0)	-1.62 (1.26)
Race/ Ethnicity (White=1; Other=0)	1.01 (1.86)
Education (At least Some College=1, No College=0)	0.84 (1.24)
Employment status (Employed Full or Part time=1, Not Employed=0)	1.72 (1.20)
Living situation (Live Alone=1; Live with Others=0)	-0.59 (1.49)
Time since diagnosis	0.67 (0.67)

*Abbreviations: SE, Standard Error; GASS = Glasgow Antipsychotic Side-Effect Scale; HRQoL, health-related quality of life; Q-LES-Q-SF = Quality of Life Enjoyment and Satisfaction Questionnaire-Short Form. Countries include Denmark, Norway, Spain, Italy, USA, Canada, and Australia. "Other" refers to "Gaining weight" and "Problems enjoying sex." * Denotes p-value significant at p <0.001.*

Table h. Quality of Life Enjoyment and Satisfaction Questionnaire Short Form (Q-LES-Q-SF) Item Descriptive Statistics, All Countries Combined by Gender and Overall

Q-LES-Q-SF Items	Females (N=234)		Males (N=198)		p-value[1]	Overall (N=432)	
	N	Mean (SD)	N	Mean (SD)		N	Mean (SD)
How satisfied have you been with your: Physical health	233	3.2 (0.9)	198	3.4 (0.8)	0.0243	431	3.3 (0.9)
How satisfied have you been with your: Mood	234	3.1 (1.0)	198	3.2 (1.0)	0.0717	432	3.1 (1.0)
How satisfied have you been with your: Work	233	2.8 (1.2)	196	3.1 (1.1)	0.0033	429	2.9 (1.1)
How satisfied have you been with your: Household activities	232	3.0 (1.1)	197	3.4 (0.9)	<.0001	429	3.2 (1.1)
How satisfied have you been with your: Social relationships	233	3.0 (1.1)	198	3.3 (1.0)	0.0213	431	3.1 (1.1)
How satisfied have you been with your: Family relationships	232	3.3 (1.0)	196	3.4 (1.1)	0.2551	428	3.3 (1.1)
How satisfied have you been with your: Leisure time activities	233	3.2 (1.1)	197	3.4 (1.0)	0.0597	430	3.3 (1.0)
How satisfied have you been with your: Ability to function in daily life	231	3.2 (1.0)	198	3.4 (1.0)	0.1085	429	3.3 (1.0)
How satisfied have you been with your: Sexual drive, interest and/or performance	232	2.7 (1.2)	198	3.0 (1.1)	0.0239	430	2.8 (1.1)
How satisfied have you been with your: Economic status	232	2.8 (1.1)	197	2.9 (1.1)	0.1255	429	2.8 (1.1)
How satisfied have you been with your: Living/housing situation	230	3.2 (1.1)	197	3.4 (1.0)	0.0299	427	3.3 (1.0)
How satisfied have you been with your: Ability to get around physically without feeling dizzy or unsteady or falling	231	3.4 (1.0)	196	3.5 (1.0)	0.3389	427	3.4 (1.0)
How satisfied have you been with your: Vision in terms of ability to do work or hobbies	232	3.3 (1.0)	198	3.3 (0.9)	0.9522	430	3.3 (1.0)
How satisfied have you been with your: Overall sense of well being	231	3.2 (0.9)	198	3.4 (0.9)	0.0572	429	3.3 (0.9)
How satisfied have you been with your: Medication	229	3.3 (1.0)	198	3.4 (0.9)	0.6344	427	3.4 (0.9)
How satisfied have you been with your: Rate overall satisfaction and contentment during past week[2]	159	3.2 (0.9)	144	3.3 (0.9)	0.1617	303	3.2 (0.9)
Total Score	234	43.0 (10.3)	198	45.8 (8.8)	0.0021	432	44.3 (9.7)

Abbreviations: SD = standard deviation. Higher scores indicate better enjoyment and satisfaction with life. The raw total score ranges from 14 to 70. Score ranges from 1, 'Very Poor' to 5, 'Very Good'. [1]P-values- One-way ANOVA test for continuous variables. Chi-square used for categorical variables. [2] Rate overall satisfaction and contentment during past week was not asked due to a programming error for EU countries.

Table i. Quality of Life Enjoyment and Satisfaction Questionnaire Short Form (Q-LES-Q-SF) Item Descriptive Statistics, All Countries Combined by Employment Status and Overall

Q-LES-Q-SF Items	Employed[1] (N=242)		Unemployed[2] (N=114)		Other[3] (N=79)		p-value[4]	Overall (N=435)	
	N	Mean (SD)	N	Mean (SD)	N	Mean (SD)		N	Mean (SD)
How satisfied have you been with your: Physical health	242	3.4 (0.9)	113	3.1 (0.9)	79	3.2 (0.7)	0.0064	434	3.3 (0.9)
How satisfied have you been with your: Mood	242	3.2 (1.0)	114	3.1 (1.0)	79	3.0 (0.9)	0.2282	435	3.1 (1.0)
How satisfied have you been with your: Work	242	3.2 (1.1)	111	2.4 (1.1)	79	2.7 (1.1)	<.0001	432	2.9 (1.1)
How satisfied have you been with your: Household activities	241	3.5 (1.0)	112	2.9 (1.1)	79	3.0 (1.1)	<.0001	432	3.2 (1.1)
How satisfied have you been with your: Social relationships	242	3.3 (1.1)	113	2.8 (1.1)	79	3.1 (1.0)	<.0001	434	3.1 (1.1)
How satisfied have you been with your: Family relationships	241	3.4 (1.1)	112	3.1 (1.1)	78	3.3 (0.9)	0.0528	431	3.3 (1.1)
How satisfied have you been with your: Leisure time activities	242	3.4 (1.0)	112	3.1 (1.1)	79	3.0 (1.0)	0.0016	433	3.3 (1.0)
How satisfied have you been with your: Ability to function in daily life	241	3.4 (1.0)	113	3.1 (1.1)	78	3.0 (1.0)	0.0010	432	3.3 (1.0)
How satisfied have you been with your: Sexual drive, interest and/or performance	241	3.0 (1.1)	113	2.6 (1.2)	79	2.7 (1.0)	0.0070	433	2.8 (1.1)
How satisfied have you been with your: Economic status	241	3.0 (1.0)	113	2.4 (1.1)	78	2.9 (1.0)	<.0001	432	2.8 (1.1)
How satisfied have you been with your: Living/housing situation	238	3.4 (1.1)	113	3.2 (1.0)	79	3.3 (0.8)	0.1657	430	3.3 (1.0)
How satisfied have you been with your: Ability to get around physically without feeling dizzy or unsteady or falling	239	3.5 (1.0)	112	3.4 (1.1)	79	3.3 (0.9)	0.5379	430	3.4 (1.0)
How satisfied have you been with your: Vision in terms of ability to do work or hobbies	241	3.4 (1.0)	113	3.2 (1.0)	79	3.2 (0.9)	0.1186	433	3.3 (1.0)
How satisfied have you been with your: Overall sense of well being	240	3.4 (0.9)	113	3.1 (0.9)	79	3.2 (0.8)	0.0220	432	3.3 (0.9)
How satisfied have you been with your: Medication	240	3.5 (0.9)	112	3.1 (1.0)	78	3.3 (0.9)	0.0017	430	3.4 (0.9)
How satisfied have you been with your: Rate overall satisfaction and contentment during past week[5]	176	3.4 (0.9)	81	3.1 (0.9)	49	3.0 (0.8)	0.0010	306	3.2 (0.9)
Total Score	242	46.3 (9.4)	114	40.9 (10.5)	79	43.0 (8.1)	<.0001	435	44.3 (9.8)

Abbreviations: SD = standard deviation. N: Number of participants in the Full Analysis Population; n (%): number and percentage of participants. Higher scores indicate better enjoyment and satisfaction with life. The raw total score ranges from 14 to 70. Score ranges from 1, 'Very Poor' to 5, 'Very Good'.[1] *Employed: Full or part time work or disabled and able to work, Student, Volunteer.*
[2] *Unemployed: Unemployed or Disabled and unable to work.* [3] *Other Employment: Retired, or Homemaker.* [4] *P-values- One-way ANOVA test for continuous variables. Chi-square used for categorical variables.* [5] *Rate overall satisfaction and contentment during past week) was not asked due to a programming error for EU countries.*
